# Unraveling the Possibilities: Recent Progress in DNA Biosensing

**DOI:** 10.3390/bios13090889

**Published:** 2023-09-18

**Authors:** Meng Yu, Tingli He, Qianqian Wang, Cheng Cui

**Affiliations:** Molecular Science and Biomedicine Laboratory (MBL), State Key Laboratory of Chemo/Biosensing and Chemometrics, College of Chemistry and Chemical Engineering, Hunan University, Changsha 410082, China; myu@hnu.edu.cn (M.Y.);

**Keywords:** DNA biosensor, aptasensor, DNAzyme, i-motif, G-quadruplex

## Abstract

Due to the advantages of its numerous modification sites, predictable structure, high thermal stability, and excellent biocompatibility, DNA is the ideal choice as a key component of biosensors. DNA biosensors offer significant advantages over existing bioanalytical techniques, addressing limitations in sensitivity, selectivity, and limit of detection. Consequently, they have attracted significant attention from researchers worldwide. Here, we exemplify four foundational categories of functional nucleic acids: aptamers, DNAzymes, i-motifs, and G-quadruplexes, from the perspective of the structure-driven functionality in constructing DNA biosensors. Furthermore, we provide a concise overview of the design and detection mechanisms employed in these DNA biosensors. Noteworthy advantages of DNA as a sensor component, including its programmable structure, reaction predictility, exceptional specificity, excellent sensitivity, and thermal stability, are highlighted. These characteristics contribute to the efficacy and reliability of DNA biosensors. Despite their great potential, challenges remain for the successful application of DNA biosensors, spanning storage and detection conditions, as well as associated costs. To overcome these limitations, we propose potential strategies that can be implemented to solve these issues. By offering these insights, we aim to inspire subsequent researchers in related fields.

## 1. Introduction

A sensor is an analytical device composed of a sensing element and a signal transducer. The function is mainly fulfilled through the specific recognition between the sensing element and the analyte, associated with one or more changes in physical and chemical properties (usually pH variation, electron transfer, uptake or release of gas or specific ions). Subsequently, the transducer converts these changes into a readable signal, typically electrical and/or optical, where the intensity or frequency change of the detection signal correlates directly with the concentration or performance of the target of interest. This process enables qualitative and quantitative analysis.

Inspired by nature, numerous researchers are actively engaged in assembling biosensors from natural or artificial biomolecules. A critical distinction between a biosensor and a physical/chemical sensor is that the former employs a biological or biomimetric substance as its sensing component. For optimal performance, biosensors need several essential characteristics: (1) High specificity and sensitivity: it should exhibit a superb ability to identify targets and analogs with high selectivity and low limit of detection (LOD). (2) Rapid response: it should be able to detect and analyze targets in a very short time frame using small amounts of sample, allowing efficient and time-effective analysis. (3) Ease of operation: the biosensor avoids long-term and multi-step operations, with relatively small size and low cost. Generally, no extensive pre-treatment of samples is required, which is convenient for on-site detection. (4) Reusability: the biosensor should be capable of testing many samples reliably, either repeatedly or continuously. This recyclability enhances versatility and cost-effectiveness in analysis.

Biosensors utilize a wide range of sensing elements, including receptors, antibodies, enzymes, nucleic acids, cell membranes [1], and cell microsomes [2]. Due to the advantages of many modifiable sites, predictable structure, high thermal stability, and biocompatibility, DNA has entered the field of vision of researchers. As the monomers comprising DNA, nucleotides contain a nucleobase (cytosine (C), guanine (G), adenine (A), or thymine (T)), deoxyribose sugar, and a phosphate group. This molecular composition endows DNA with rich interaction forces, such as hydrogen bonds, electrostatic adsorption, hydrophobic interactions, π–π stacking, and dipolar forces. By artificially synthesizing and modifying nucleotide monomers, the potential for DNA to interact with other substances can be expanded. Moreover, the cost of DNA synthesis is relatively low, and batch-to-batch variation is minimal, increasing the prospects of commercialization. Meanwhile, the secondary and tertiary structures of DNA are predictable according to base pairing rules (A with T and C with G) and the relatively rigid 3D configuration of the double helix, further augmenting the utility of DNA in biosensing applications. These properties lead to wide use of DNA as both a sensing element and a transducer to detect a variety of analytes.

There have already been many outstanding reviews in this field, and most of them primarily classified biosensors based on their output signal types (optical signal, electrical signal, magnetic signal [3], etc.). Therefore, the content of these reviews emphasizes on the detection mechanisms and practical applications, but not on the recognizing elements. Each type of biosensor carries distinct advantages: electrochemical biosensors have a low detection limit and facile miniaturization; optical biosensors exhibit resistance to electronic or magnetic interference in signal changes; and piezoelectric sensors enable the evaluation of dynamic affinity interactions without requiring specific reagents [4]. These varied methodologies have their own advantages and provide researchers with ideas of innovative signal processing and reading.

**Scheme 1:**
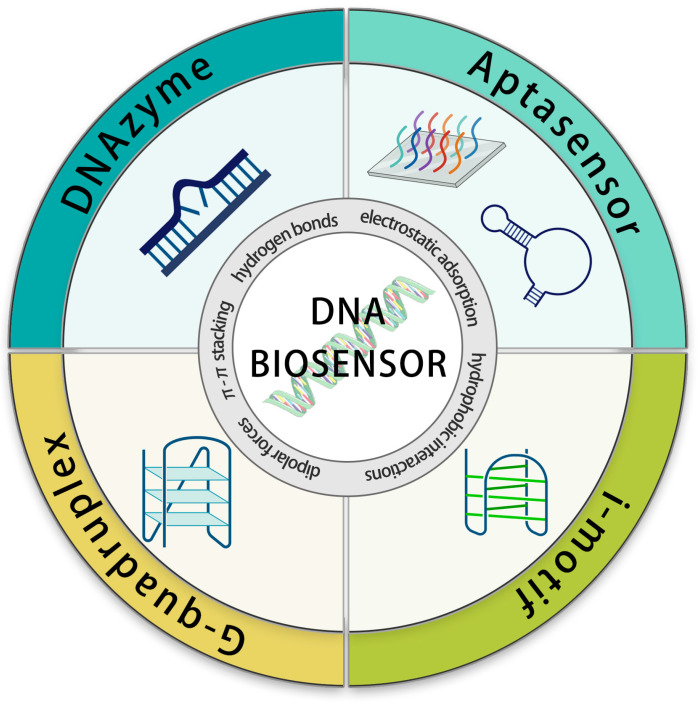
Schematic diagram of DNA biosensors based on DNA species.

Our review relies on the sensor’s detection element—the DNA structure—as the basis for classification (Scheme 1). The diverse range of DNA structures, spanning from single-stranded nucleic acids to DNA nanoparticles, imparts a spectrum of functions to DNA. Therefore, in this review, we discuss the sensing applications of DNA from a structure-driven function perspective. The variability in DNA structure can lead to the creation of more superior sensor elements. The combination between distinct element designs and signal output techniques yields an array of analytical methods that enables biosensors to work across diverse detection environments and purposes. In this review, we explore the cutting-edge advances in the highly promising field of DNA sensing. We categorize these advancements based on the types of DNA species and introduce their detection mechanisms, highlight recent applications, and unravel the exciting possibilities and opportunities that lie ahead in DNA sensing.

## 2. DNA Sensing Methods and Their Applications

### 2.1. Sensing with Aptasensors

Aptasensors utilize aptamers as the recognition unit and convert the recognition events into electrical or optical signals. Aptamers are single-stranded oligonucleotides (DNA or RNA) with high affinity and specificity to targets due to their specific three-dimensional structures and intermolecular attractions [5,6,7]. Numerous studies have shown that aptamers can specifically recognize cells [8], proteins [9], antigens [10], nucleic acids, and metal ions [11,12]. As nucleic acids, aptamers have several intrinsic benefits such as excellent thermal stability, cost-effectiveness, minimal immunogenicity, low batch-to-batch variation, and high designability.

**Figure 1:**
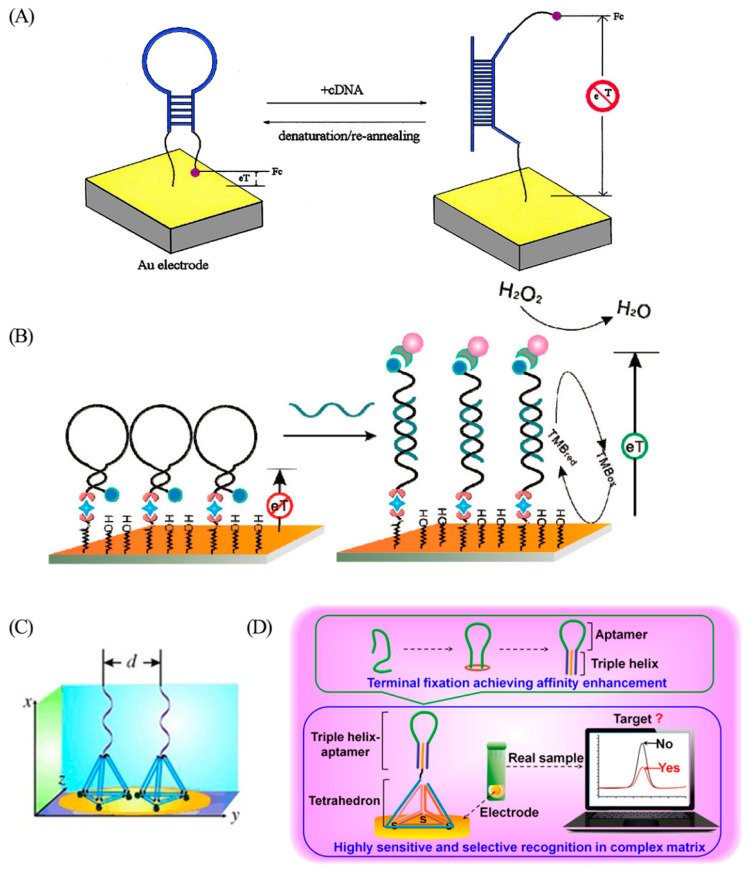
(**A**) Scheme of the ferrocene-based E-DNA sensor. Reprinted with permission from [13]. Copyright (2003) National Academy of Sciences, U.S.A. (**B**) Scheme of the enzyme-based E-DNA sensor. Reprinted with permission from [14]. Copyright (2008) American Chemical Society. (**C**) Schematic diagram of the third generation of E-DNA sensor. Reprinted with permission from [15]. Copyright (2015) WILEY-VCH Verlag GmbH and Co. KGaA, Weinheim. (**D**) Scheme of the termini-fixed aptamer sensor. Reprinted with permission from [16]. Copyright (2019) American Chemical Society.

Since their invention in 2003, a series of electrochemical (E)-DNA sensors has been developed by the Fan group. Electrochemical sensors have attracted significant attention due to their advantages, such as low cost, simple structure, easy miniaturization, high selectivity, excellent sensitivity, and good stability. Fan et al. combined a stem-loop DNA structure with a ferrocene tag and immobilized it on the surface of a gold electrode for the detection of specific DNA sequences (Figure 1A) [13]. In the absence of the target sequence, the proximity of the ferrocene to the electrode surface promotes fast electron transfer and efficient redox of ferrocene. Upon hybridization with the target sequence, the redox current changes because the ferrocene moves away from the electrode surface. With an LOD of around 10 pM, E-DNA sensors have potential advantages, both in detecting DNA in solution and in complex systems such as serum. Even with a target DNA concentration of 10 fM, second-generation E-DNA, with ferrocene replaced by an enzyme, can still clearly detect DNA (Figure 1B) [14].

Nevertheless, the stem-loop probe occasionally exhibits insufficient rigidity and spontaneous unfolding due to the influence of surface occupancy, potentially leading to false positive signals. To address these issues, the third generation of E-DNA was designed as a DNA tetrahedron with a stem-loop probe protruding from the top (Figure 1C) [15]. This tetrahedral structure ensures that the DNA recognition probes are oriented upwards, and the distance between the stem and loop structures is precisely controlled by the tetrahedron’s side length. The intertwining between the stem-loop probes is avoided to ensure the stability of the probe’s structure, thereby improving the target hybridization efficiency and leading to reduced background noise. To further amplify recognition affinity, Zhao et al. proposed a triple-helical end-fixed aptamer design to stabilize the conformational structure (Figure 1D) [16], leading to an LOD 180 times lower than that of the end-fixed aptamer. These advancements in the design and optimization of E-DNA sensors have demonstrated their substantial potential for highly sensitive and selective DNA detection.

**Figure 2:**
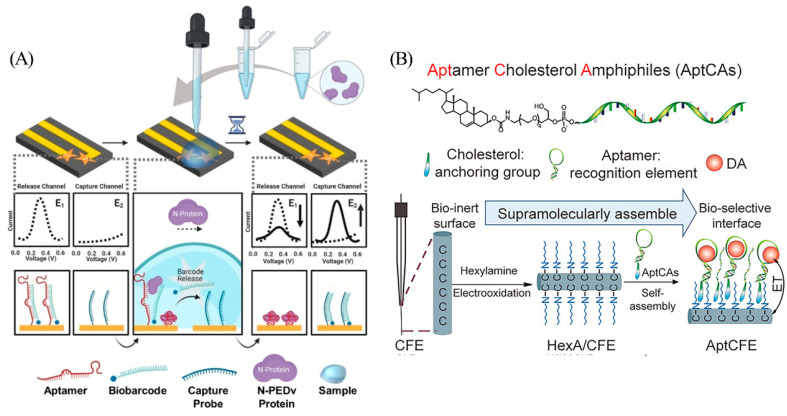
(**A**) Components and principle of DEE-Chip assay. Reprinted with permission from [18]. Copyright (2022) Wiley-VCH GmbH. (**B**) Schematic of aptCFE sensor for dopamine. Reprinted with permission from [20]. Copyright (2020) Wiley-VCH GmbH.

Attaching redox labels directly to the aptamer usually generates a large background signal, which poses a challenge for sensitive detection. Designs that rely on strand displacement for signal transduction present a promising alternative approach [17]. In order to reduce the background signal, Li et al. designed a dual-electrode electrochemical chip (DEE-chip) composed of a detection probe (consisting of aptamer named “PEA1–3” and a partially complementary strand named “biobarcode”) on one electrode and a capture probe on another electrode (Figure 2A) [18]. After PEA1–3 binding to the target, the biobarcode falls off and is bound to the capture strand on the adjacent electrode to form a new double-stranded product. The voltage difference between the two electrodes can be detected if target binding events occur. Target acquisition and signal reporting on separate electrodes facilitates signal-on sensing mechanism while minimizing background noise.

Apart from the electrode surface, aptamers can be immobilized onto nanomaterials to form different biosensors. Gold nanoparticles exhibit several advantageous attributes: excellent biocompatibility and stability, facile synthesis, tunable size, and widespread utility in bionanotechnology. Furthermore, gold nanoparticles can provide a large specific surface area, facilitating the modification of diverse small molecules while preserving their biological activity. One study combined aptamers with a 3D gold nano-microisland microfluidic platform for highly selective and sensitive parasite detection. Even after 21 days of freezing, the sensor’s current reached 85% of the initial current effect. The sensor shows remarkable stability and is expected to be a promising candidate for point-of-care testing (POCT) application in parasite detection [19].

Hou et al. designed an aptCFE sensor for dopamine monitoring in living rat brains (Figure 2B) [20]. This device utilizes a non-covalent cholesterol-alkyl chain interaction to effectively fix aptamers to in vivo compatible carbon fiber microelectrode (CFE) surfaces with clinically relevant LOD as low as 6 nM. This strategy is highly specific, anti-fouling, and programmable, demonstrating excellent spatio-temporal resolution in the detection of dopamine in vivo.

**Figure 3:**
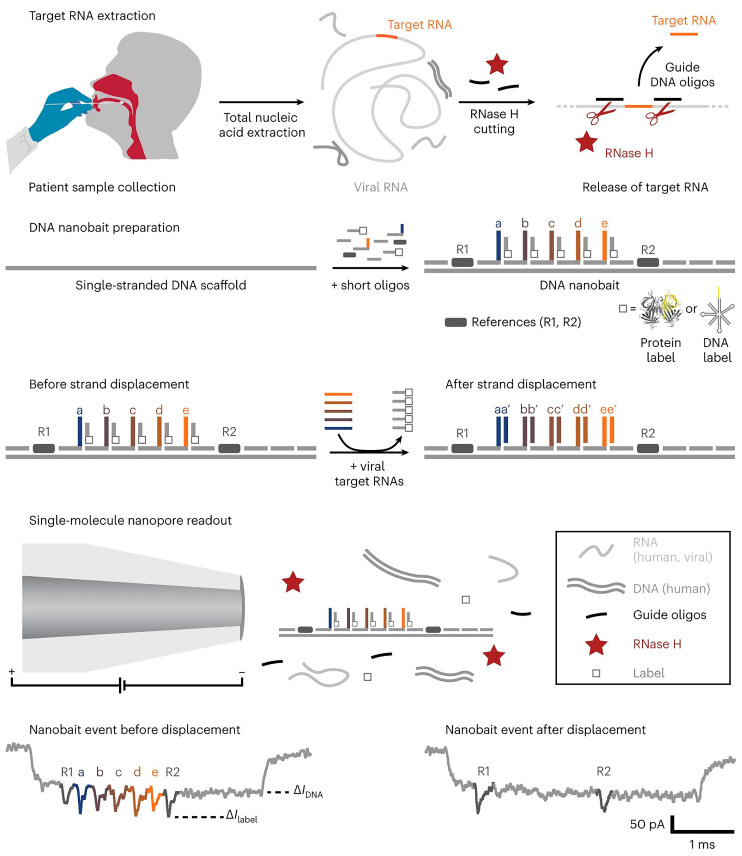
Scheme of the nanobait assay. Reprinted with permission from [21]. Copyright (2023) Springer Nature.

Based on nanopore sequencing technology, Bošković et al. created a self-assembled DNA nanobait that can simultaneously recognize multiple short RNA targets via toehold-mediated strand displacement (Figure 3) [21]. Nanobait incorporates five distinct and specific binding sites corresponding to the five different short target RNAs to be detected. Each nanobait is voltage driven through the nanopore and detected in a molecular mixture, with each tag exhibiting a specific current fluctuation. Upon interacting the intended target, the corresponding tag chain undergoes replacement and the current pattern changes. Compared with the initial current signal, it is possible to rapidly and viably distinguish which target RNA is present. The authors have successfully utilized the nanobait to precisely determine the presence or absence of SARS-CoV-2 in patient swabs.

**Figure 4:**
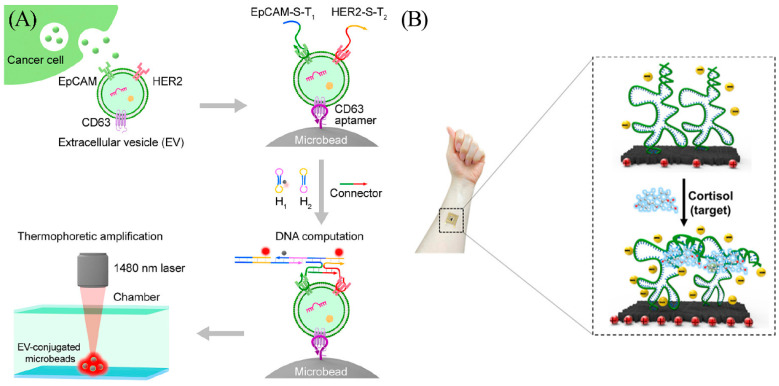
(**A**) Principle of the thermophoresis-mediated DNA computation on EV membranes. Cancer cells release EVs (green dots), and aptamers can recognize proteins on the EV membranes. This would trigger the HCR and generate detectable fluorescent signals (red dots). Reprinted with permission from [23]. Copyright (2021) American Chemical Society. (**B**) Scheme of the wearable cortisol aptasensor. Reprinted with permission from [25]. Copyright (2022) American Chemical Society.

Thermophoresis is the directional movement of particles induced by a temperature gradient [22]. Changes in the mass, charge, hydration layer, and conformation of biomolecules can all bring variations in the rates of motion of molecules in the temperature gradient field and therefore can be used for isolation of certain targets from complex systems. For this reason, thermophoresis has gained considerable attention in recent years. Recognizing the potential of thermophoresis, Li et al. combined it with DNA logic gate to develop a sensing method for tumor-derived extracellular vesicles (tEVs) (Figure 4A) [23]. The authors amplified the signal with the assistance of hybridization chain reaction (HCR) and were able to distinguish breast cancer patients from healthy donors in a clinical cohort (n = 30) with 97% accuracy. HCR is a DNA-triggered amplification method proposed by Pierce et al. [24]. To identify breast cancer with enhanced accuracy, the author used two aptamers to sense two different biomarkers on tEVs. HCR occurs only when both biomarkers are present. As a result, stem-loop hybridization and opening reactions occur continuously to form long double-stranded structures. The tEVs are then accumulated by thermophoretic accumulation, thus amplifying the fluorescence signal and enhancing detection sensitivity.

Wearable device technology enables real-time perception of the wearers and their surroundings. The advancement of disease detection has shifted the concept of protecting human health from hospital-centric diagnostics to patient-centric diagnostics. This change has greatly fueled the development of the wearable device market. An et al. proposed a flexible and portable aptasensor for feasible and rapid real-time monitoring of cortisol that remains highly selective with a detection limit of 10 pM (response time was less than 5 s). In addition, the device performs well in detecting the presence of analyte even when the concentration of the interfering substances is 100 times that of the analyte (Figure 4B) [25]. The cortisol aptasensor is able to detect around 1 nM cortisol from real sweat within minutes.

### 2.2. Sensing with DNAzymes

Deoxyribozymes, also known as DNAzymes, are DNA oligonucleotides capable of performing a specific biochemical reaction via catalytic functions [26]. Their modular structure consists of two essential parts, a central catalytic core and two substrate-binding arms. DNAzymes with specific catalytic activity on the substrate of interest can be obtained from billions of DNA candidates by Systematic Evolution of Ligands by Exponential Enrichment (SELEX) [27].

DNAzymes can cyclically catalyze a reaction in the presence of metal ions, which is conducive to the design of sensor structures for signal amplification. Featuring facile synthesis, high sensitivity, distinct specificity, and signal amplification, DNAzymes are highly versatile in the detection of metal ions, nucleic acids, and bacteria. Furthermore, DNAzymes have been successfully combined with aptamers [28] and nanomaterials [29,30] for the development of multiplex bioassay.

Using DNAzymes as sensors can offer several advantages: (1) Utilizing nucleic acids as biocatalysts can mitigate non-specific adsorption of proteins at nucleic acid interfaces or electrode surfaces. This challenge of non-specific adsorption is often encountered in biosensing, and the aid of non-complementary negatively charged nucleic acid tags can alleviate this shortcoming. (2) Base sequences within nucleic acids, including DNAzyme catalytic units and substrate-binding arms, are programmable, allowing for the precise design and customization of sensor properties.

Metal ions are essential for living cells. For instance, iron and manganese serve as important cofactors in DNA replication and repair, while zinc acts as a structural element in protein organization and plays a crucial role in intracellular and intercellular communication. Dysregulation of copper has been demonstrated to have implications for cancer and neurodegeneration [31,32,33]. Deviation from the standard range of metal ion concentrations can significantly impact the physiological functions of the human body. The need to monitor the concentration of metal ions for the prevention and treatment of disease has accelerated the development of detection technology.

**Figure 5:**
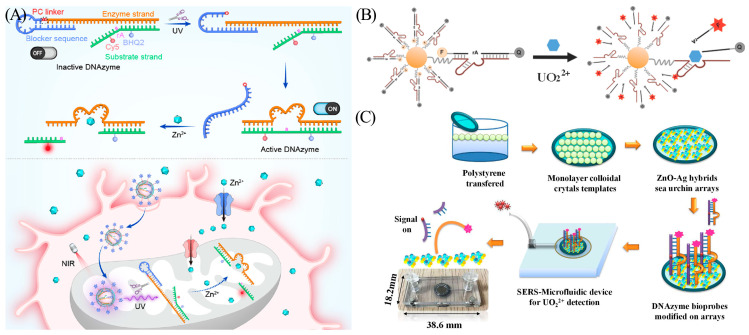
(**A**) Components of L-DZ and the mechanism of L-DZ/mUC for spatial imaging. Reprinted with permission from [35]. Copyright (2022) American Chemical Society. (**B**) Schematic diagram depicting the DNAzyme-AuNP sensor for the detection of UO_2_^2+^. Reprinted with permission from [37]. Copyright (2013) American Chemical Society. (**C**) Scheme of the SERS-based microfluidic biosensor. The red dots represent UO_2_^2+^. The pink dots represent Raman reporting group RhB. Reprinted with permission from [38]. Copyright (2020) Elsevier.

Yi et al. designed a DNA nanodevice, L-DZ/mUZ, that facilitated mitochondria-specific sensing and imaging of Zn^2+^ in living cells (Figure 5A) [34]. This nanodevice consists of an ultraviolet (UV) light-activatable DNAzyme sensor whose catalytic chain is blocked by a photocleavable linker (PC linker) sequence. When the PC linker bond is cleaved under UV light irradiation, the DNAzyme is released, and the activated sensor recognizes zinc ions in mitochondria ([Zn^2+^]_m_) and generates a fluorescence signal. In addition, to confirm the imaging capabilities of the nanodevice in vivo, the authors created an injury cell model of Zn^2+^ toxicity and a drug treatment model for ischemia. L-DZ/mUC was successfully applied for the monitoring and assessment of dynamic [Zn^2+^]_m_ changes. In order to make DNAzyme more suitable for metal ion sensing, a study showed that the catalytic activity of the F8 DNAzyme, which specifically recognizes Cu (II), can be significantly improved by the addition of polydopamine and gold nanoparticles [35].

Heavy metal ions are prominent environmental pollutants. They persist without degradation and can accumulate in the food chain, generating serious damage to the ecosystems and endangering human health. Even trace amounts of uranyl ions (UO_2_^2+^) can cause irreversible damage to the human body. The UO_2_^2+^-specific DNAzyme was first designed by the Lu group in 2003 [36]. The DNAzyme sensor features high sensitivity with a detection limit of 45 pM and a dynamic range of 1 to 400 nM. Wu et al. first developed a DNAzyme-AuNP (gold nanoparticle) probe for the detection of UO_2_^2+^ in living cells (Figure 5B) [37]. After identifying uranyl ions, the DNAzyme cleaves the substrate chain, leading to the recovery of the initially quenched fluorophore as the AuNP quencher dissociates.

However, detecting fluorescence typically requires large laboratory instruments and professional operators, making them unfeasible for emergency field testing. To address this challenge, a new approach was presented by He and his co-workers. The authors developed an ultrasensitive and recyclable surface-enhanced Raman spectroscopy (SERS) microfluidic UO_2_^2+^ biosensor (Figure 5C) [38]. After interaction with UO_2_^2+^, the DNAzyme-cleavage reaction is initiated. A strong Raman signal is generated after the substrate chain is deposited on the surface of the SERS substate. The deposited substrate chain induces a “memory effect” on the channel surface, thereby affecting the sensitivity and reproducibility. Notably, the biosensor can be reused more than three times by replenishing the DNAzyme substrate chain and cleaning the microfluidic device. This overcomes the SERS single-detection limitation described in previous reports [38]. The introduction of SERS microfluidic technology reduced the LOD to 0.72 pM, approximately five orders of magnitude lower than the value set by the Environmental Protection Agency (EPA) for drinking water quality.

Scientists first identified microRNAs (miRNA) in 1993 [39]. These short non-coding RNAs are expressed in almost all eukaryotes and even several viruses [40]. Numerous studies have proven that miRNA downregulates the expression of its target messenger RNAs (mRNAs) by forming an RNA-induced silencing complex (RISC) associated with argonaute (Ago) family proteins. According to the central dogma, mRNAs are translated by ribosomes into synthetic proteins that are relevant to numerous cellular signaling pathways and biological functions. Apparently, miRNA biology is an intricate and highly coordinated pattern of gene regulation that can affect almost all biological processes in mammals and plays a particularly essential role in cardiovascular diseases [41], cancers [42,43,44], and neurological disorders [45,46].

**Figure 6:**
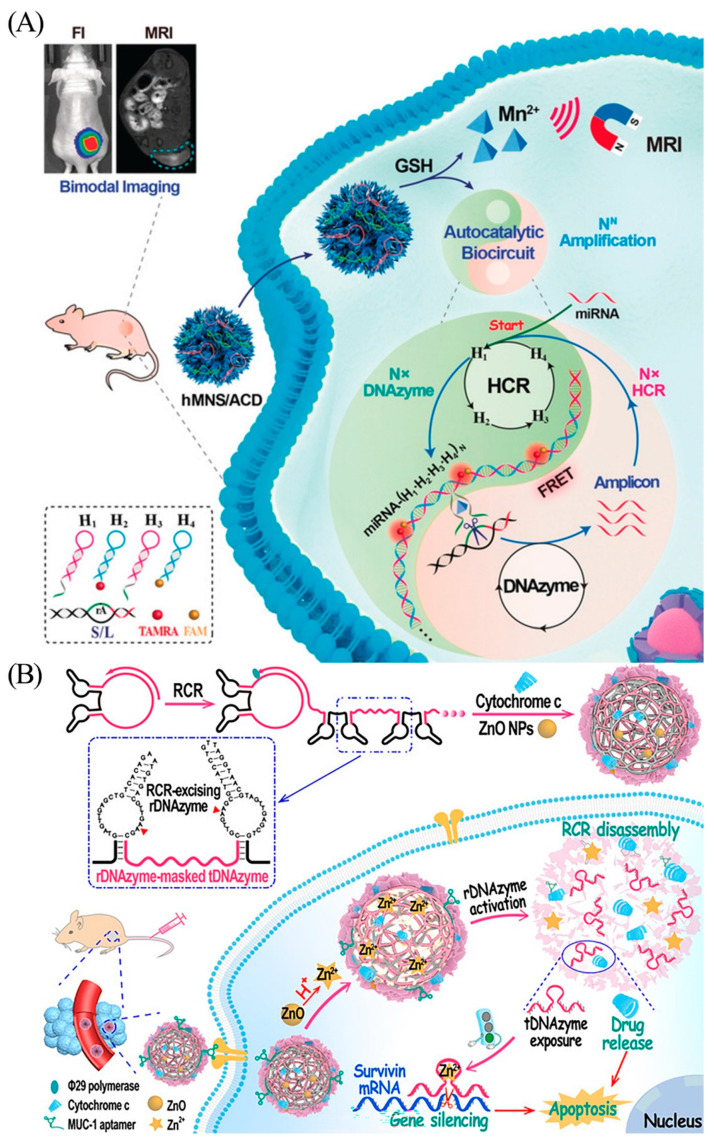
(**A**) Scheme of the DNAzyme biocircuit for in vivo amplified miR-21 imaging. Reprinted with permission from [49]. Copyright (2020) Wiley-VCH Verlag GmbH and Co. KGaA, Weinheim. (**B**) Preparation of multifunctional self-catabolic bi-DNAzyme nanoparticle and its cascade in cancer cells. Reprinted with permission from [50]. Copyright (2021) Wiley-VCH GmbH.

Consequently, swift and sensitive miRNA measurement for early disease screening is extremely important. Current miRNA detection methods [47] include microarrays; RNA sequencing; and reverse transcriptase real-time quantitative PCR (qRT-PCR), which is considered as the gold standard for miRNA testing. However, qRT-PCR tends to be expensive and requires specialized expertise, which limits its application in cancer detection. Moreover, conventional qRT-PCR can yield false-negative results even with trace amounts of free nucleic acids [48]. Therefore, an innovative approach, which is inexpensive, easier to perform, and reliable, is necessary to improve the accuracy of miRNA detection. Wei et al. chose endogenous miRNA as the initiator chain to activate HCR by linking multiple DNAzymes to generate nanowires (Figure 6A) [49]. The synergistic activation of the HCR reaction and DNAzyme biocatalysis promotes precise signal localization and signal amplification of trace miRNAs in vivo. Wang et al. designed a self-catabolic bi-DNAzyme nanoparticle (Figure 6B) [50]. Hydronium ions react with ZnO to form Zn^2+^, leading to a collection of activation cascades. Activation of the rDNAzymes (stimuli-responsive DNAzymes) decomposes the nanoparticles, thereby releasing the tDNAzymes (therapeutic DNAzymes) against surviving mRNA, as well as the drug to promote gene silencing and induce apoptosis.

**Figure 7:**
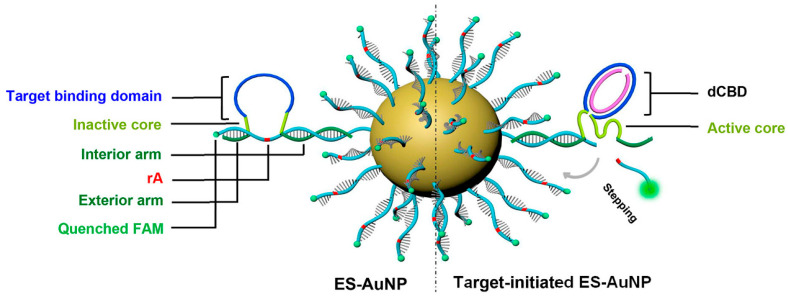
Design of ES-AuNP nanoprobe. Reprinted with permission from [51]. Copyright (2021) American Chemical Society.

Gao et al. designed ES-AuNP (Figure 7) [51], a self-protected DNAzyme walker for intracellular miRNA imaging, which also provides a simple but efficient tool for trace miRNA detection. The target-binding domain (single-stranded semicircular bulging DNA, sCBD, also called walker chain) of ES-AuNP detects the target miRNA. With the help of the cofactor magnesium ion, DNAzyme forms a catalytic structure and cleaves rA (adenosine) from the substrate chain with FAM label, and fluorescence is restored. The toe-mediated chain replacement reaction triggers the walker to “walk” to the next substrate chain, and it amplifies the fluorescence signal. DNAzyme walker demonstrated a fivefold increase in serum resistance and an eight-fold increase in catalytic activity compared to commercial miRNA testing reagents and FISH technology. By changing the target binding domain, the DNAzyme walker can be adapted to other miRNA imaging. With a similar design to ES-AuNP, in order to avoid the unstable complementarity between DNAzyme and substrate strand, Yin et al. linked DNAzyme directly to gold nanoparticles [52]. Zhao et al. also used nanoamplification technology to design a 3D nanomaterial capable of detecting microRNA-155 concentrations at the attomolar level [53]. The AuNPs/GAs/CFP as sensor substrates have good conductivity and offer a larger specific surface area, which allows more DNAzyme-linked magnetic beads to bind to the nanomaterials. As a result, the biosensor exhibited a relatively low LOD of 56.23 aM with a linear range of 100 aM to 100 nM.

DNAzymes have also demonstrated the capacity to recognize bacteria. Conventional methods for detecting bacteria include bacterial culturing [54], enzyme-linked immunosorbent assay (ELISA) [55], and polymerase chain reaction (PCR) technology [56]. However, these methods are laborious, muting sensitive, costly, and not appropriate for on-site applications [57,58]. The most commonly used non-invasive test for pathogenic bacteria is the urea breath test (UBT), which relies on urease detection. However, since other bacteria also produce urease, the specificity is inadequate. Therefore, some researchers have directed their attention towards low-cost and sensitive DNAzyme-based assays.

**Figure 8:**
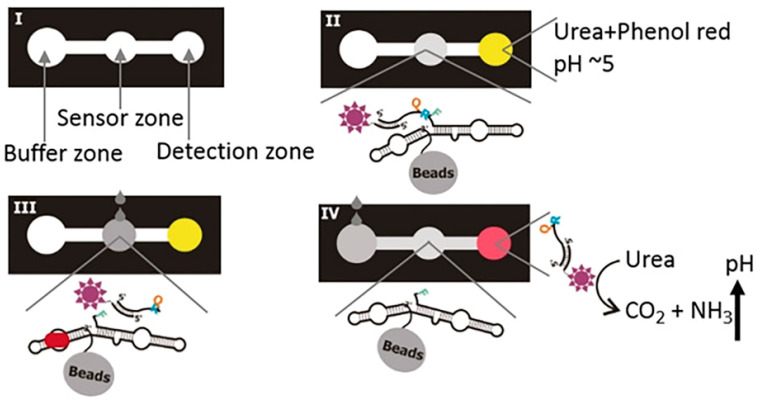
Components and principle of litmus test strips for HP detection. (**I**) The three zones of the test strip. (**II**) The central zone is the DNAzyme sensor, and the detection zone on the right serves as the signal output component. (**III**) Sample drops activate DNAzyme, thereby releasing urease. (**IV**) Flowing buffer is added to the buffer zone, causing the urease molecules to move towards the detection zone to hydrolyze urea and produce ammonia, which induces a color change to red. Reprinted with permission from [60]. Copyright (2019) Wiley-VCH Verlag GmbH and Co. KGaA, Weinheim.

In 2014, the Li group first translated the molecular recognition signal produced by an aptazyme into a pH change [59]. Subsequently, Ali et al. produced litmus test strips containing DNAzyme that can be used to detect *Helicobacter pylori* (HP) (Figure 8) [60]. The entire test strip consists of three parts: the sensor zone, which contains the DNAzyme molecule bound to urease; the right detection zone, containing urea and phenol red; and the left buffer zone with the running buffer. The sample is placed in the sensor zone and, when HP is recognized by the DNAzyme, a cleavage reaction occurs to release fragments containing urease. At the same time, free urease is delivered by the buffer to the detection zone to hydrolyze the urea, and the generated ammonia changes the litmus from yellow to red. This detection method can detect at least 104 colony-forming units (CFU) per milliliter, similar to the solution-based assay. Furthermore, these paper strips can be stored at room temperature for nearly 4 months, which makes the concept of conducting bacterial tests in households and by individuals more realistic. However, litmus paper can provide only a qualitative result, which limits the application.

Pandey et al. reported an electrochemical analysis method using electroactive RNA-cleaved DNAzymes (e-RCDs) to identify specific bacterial targets. DNA barcodes are released and transmit the signal to an electronic chip, which displays the results on smartphones [61]. Like the DEE-chip mentioned above, e-RCDs greatly reduce the background signal. At the same time, the dendritic “star” nanocarrier provides many sensor-fixing sites. The results from 41 clinical samples attained a diagnostic sensitivity of 100% and a specificity of 78% in less than one hour of analysis time. With advances in electronic chip fabrication and miniaturization of biosensor platforms, the authors believe that this design can be assembled into a complete, reagent-free POCT kit capable of rapid test against various pathogens.

### 2.3. Sensing with i-Motif

In 1993, Leroy and Guéron reported the formation of a four-stranded intercalated structure through the association of cytosine-rich DNA sequences under acidic conditions, which became known as the i-motif [62,63]. The i-motif sequence exists in vivo, particularly in regions of the genome associated with gene expression regulation, such as telomeres [64,65], centromeres [66], and promoter regions of proto-oncogenes [67,68].

Because of its ability to make conformational alterations in response to changes in pH, the i-motif was believed to be promising as candidate for applications in measuring intercellular pH variations. Even small changes in pH can have different effects on cells, and improved detection of these variances can lead to better understanding of intracellular or intercellular communication and related biological functions.

**Figure 9:**
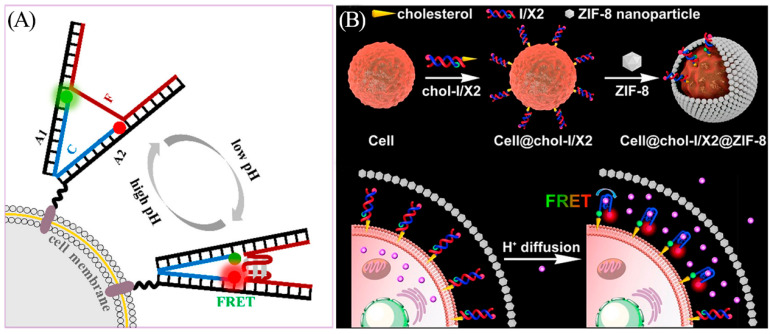
(**A**) Construction and principle of the pH-responsive DNA tweezer. Rhodamine red (red dot) and rhodamine green (green dot) connected to DNA tweezer as signal reporters. Reprinted with permission from [70]. Copyright (2018) American Chemical Society. (**B**) Schematic illustration of MOFC-i for monitoring subtle deviations in cell surface pH. Pink dots represent H^+^. Reprinted with permission from [71]. Copyright (2021) American Chemical Society.

The extracellular pH, especially within tissues and tumor microenvironment, is key to physiological and pathological processes of cells. For example, the pH of the tumor microenvironment is generally acidic compared to the healthy cellular environment due to hypoperfusion, hypoxia, inflammation, and glycolytic cellular metabolism [69]. Zeng et al. reported a DNA nanotweezer system to monitor extracellular pH (Figure 9A) [70]. The nanotweezer responds rapidly and reversibly to extracellular pH values in the range of 5.0–7.5 and exhibits high cell membrane insertion efficacy and low cytotoxicity, allowing real-time imaging of cell surface pH changes. Yang et al. proposed a biocompatible MOFC-i strategy (Figure 9B) [71]. The i-motif is inserted into the cell membrane, while the ZIF-8 nanoparticles form a biocompatible MOF shell around the cell. This strategy limits the diffusion of the secreted protons and prevents their neutralization in the extracellular matrix. The MOFC-i strategy is capable of sensitive and dynamic monitoring subtle deviations in cell surface pH, and it is expected to significantly advance pH-related diagnosis and drug screening research.

Intracellular and extracellular pH values have major impacts on a variety of cellular processes. The pH value serves as a critical reference for lysosome maturation. This is closely related to endocytosis, which depends on various attributes of the internalized substance, including its size, nature of material, and cell type [72]. After the substances attach to the plasma membrane, the invagination of the plasma membrane forms a vesicle. As the endosome matures, the internal environment of the vesicle undergoes a transformation from neutral to acidic pH. Late endosomes are able to fuse with lysosomes, which contain a highly acidic environment of pH 5.0 to 4.5 and are optimal for the activity of degradative enzymes (lysosomal hydrolases). Impaired endocytosis may have carcinogenic potential [73]. Because the pH of lysosomes varies slightly at different stages of maturation, it is of interest to researchers to accurately map the different stages of lysosome formation.

**Figure 10:**
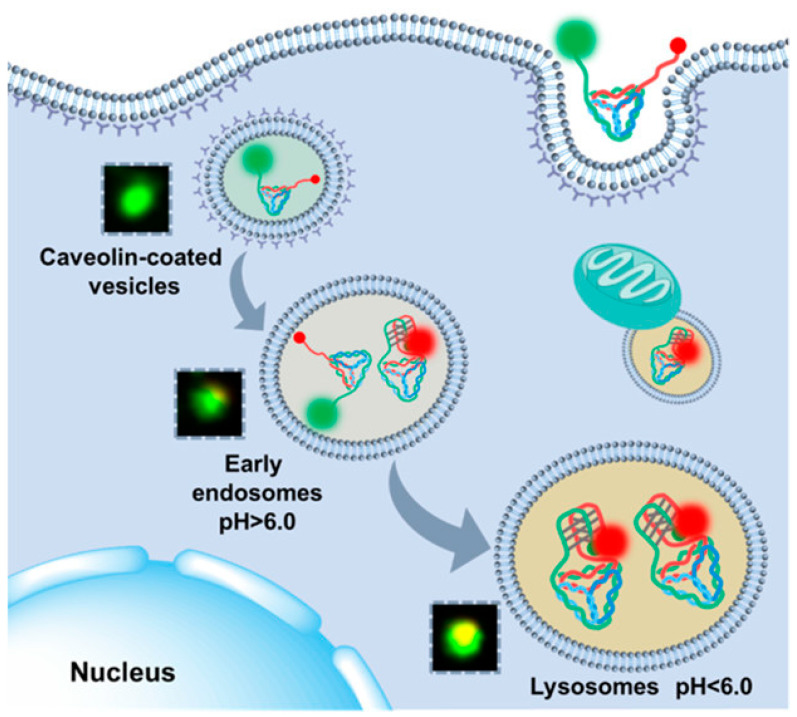
Schematic diagram of the process of DNA tetrahedron tracking lysosomes and detection of lysosomal pH fluctuations. Reprinted with permission from [77]. Copyright (2021) American Chemical Society.

However, there are some challenges associated with using i-motifs for intercellular applications, especially lysosome sensing. Single-stranded DNA is easily degraded by nucleases, which limits its use in certain applications. To address this problem, researchers have been exploring new methods to stabilize the i-motif structure, for example, by using the i-motif as extended strands of the DNA tetrahedron or as part of the DNA tetrahedron framework [74]. He et al. found that the i-motif branches co-programmed with DNA frameworks are more sensitive in detecting small pH fluctuations, offering the potential for lysosome analysis [75]. A study has shown that changes in the pH detection range and pH transition midpoint of the i-motif can be achieved by changing its sequence and the continuous number of cytosines [76]. Yue et al. (Figure 10) [77] split the i-motif into two parts as the branches of a DNA tetrahedron with side length as the distance between the two split parts. This design was successfully applied in response to pH at different maturation stages of lysosomes.

Liu et al. used the i-motif as the backbone of a DNA framework [78], which acted as a dynamic pH-response machine (DFN). The stretched and contracted forms of the DFN display different colors at different pH values. This nanomachine successfully analyzed the dynamic coloration of pH changes within vesicles during endocytosis and exocytosis, facilitating the study of vesicle fusion kinetics. This nanomachine will potentially serve as an efficient choice for dynamic coloration sensing in artificial cells in the future.

### 2.4. Sensing with G-Quadruplexes

The G-quadruplex (G4) is a three-dimensional secondary structure of nucleic acids formed by guanine-rich strands through intramolecular or intermolecular Hoogsteen hydrogen bonds [79,80,81]. Like other functional nucleic acids, G4 demonstrates the advantages of high stability and ease of chemical modification [82]. Resembling long quadrilateral hollow tubes with periodic “pockets”, the quadruplexes have a central carbonyl-lined channel that can accommodate alkali metal ions, and the presence of potassium ions has been reported to further stabilize the structure of the G-quadruplex [81,83,84]. Therefore, G4 has been widely used as the molecular recognition unit in biosensors for metal ions [85].

The G4 can interact with various substances, including porphyrins [86] and some organic dyes [87]. Porphyrins, such as N-methylmesoporphyrin IX (NMM), have a planar aromatic structure, and some of them can interact with G-quadruplex bodies via π–π stacking [88]. The combination of organic dyes with G-quadruplexes enhances the structural rigidity and planarity of the organic molecules, resulting in enhanced fluorescence of organic dyes, while the complexes also effectively stabilize the G-quadruplex structure [89,90]. Therefore, G-quadruplexes can be used as fluorescent probes for the construction of DNA biosensors.

On the other hand, G-quadruplexes bind to heme chloride (hemin), an important cofactor of many enzymes, to form G-quadruplex/hemin complexes [91]. The G-quadruplex can stabilize hemin via π–π stacking and greatly enhance its catalytic activity, resulting in a G-quadruplex/hemin with peroxidase activity [92]. Similar to horseradish peroxidase (HRP), the detection of analytes can be realized by catalyzing substrate oxidation, for example, by hydrogen peroxide, to make DNA sensors that produce color changes, electrical signals, or chemiluminescence signals [93]. Furthermore, compared to HRP, the G-quadruplex/hemin complex is an artificial enzyme with several advantages, such as compact size, facile synthesis and manipulation, high stability, and economical production [94].

Strontium-90 (Sr-90) is one of the radionuclides produced by the fission of uranium and is classified as a class I carcinogen with a half-life of 29 years [95]. Due to its chemical similarity to calcium, Sr-90 tends to accumulate in human bones and can cause bone tissue sarcomas and even leukemia [96]. Radioactive Sr-90 has been detected in soil, water, and even living organisms [97]. Considering the health issues it may cause to human beings, the detection of Sr^2+^ is of great importance. Utilizing the property that Sr^2+^, like K^+^, can induce G-rich sequences to form quadruplex secondary structures [98], Leung et al. developed a G-quadruplex selective luminescent iridium (III)-containing metal complex probe for the selective detection of Sr^2+^ in buffer solutions and seawater [99]. They demonstrated the feasibility of using G-quadruplexes for Sr^2+^ measurement but could not fulfill the requirements for trace Sr^2+^ detection in the environment. In 2023, Feng et al. designed a signal-off biosensor, TiG4-DNA, based on the fluorescent molecule Thioflavin T (ThT), which can induce formation of a G-quadruplex [100]. Due to the higher binding affinity to G-quadruplexes than ThT, Sr^2+^ can release ThT from TiG4-DNA, resulting in weakened fluorescence intensity. This design is highly selective for Sr^2+^ with an ultralow detection limit of 2.11 nM.

**Figure 11:**
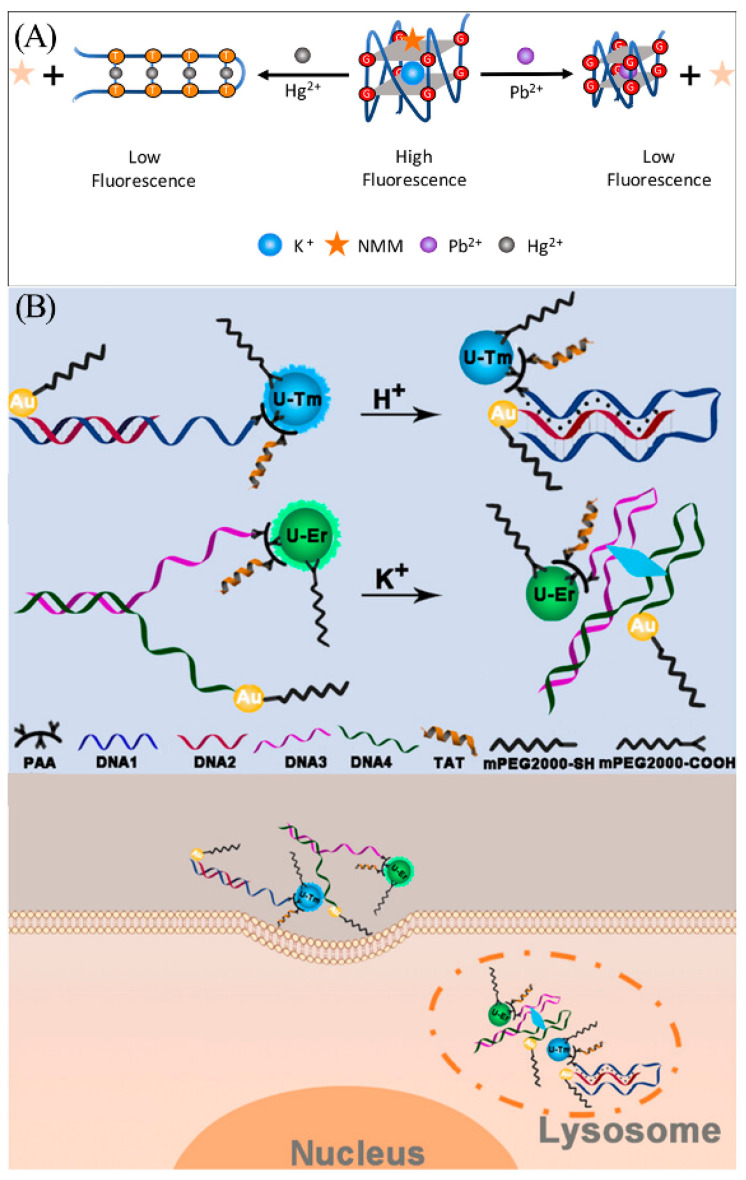
(**A**) Schematic illustration of K^+^-induced G-quadruplex/NMM fluorescent probe for detection of Hg^2+^ and Pb^2+^. Reprinted with permission from [104]. Copyright (2018) Elsevier. (**B**) Construction and principle of nanosensors for detection of K^+^ and H^+^ in lysosomes. Reprinted with permission from [105]. Copyright (2020) Wiley-VCH GmbH.

While heavy metal ions such as Pb^2+^ and Hg^2+^ are not radioactive, they can still inflict irreversible damage to humans. A single common battery containing Pb^2+^ and Hg^2+^ has the potential to contaminate 600,000 L of water [101,102], and these toxic metal ions can enter the body and accumulate in organs via drinking water, skin contact, and the food chain, leading to chronic poisoning [103]. Thus, it is also of great necessity to detect harmful heavy metals such as Pb^2+^ and Hg^2+^ in the environment. Zhou et al. found that the oligonucleotide AGRO100 contains many G bases and T bases and can bind selectively to Pb^2+^ or Hg^2+^. Based on this property, the team constructed a bifunctional K^+^-induced G-quadruplex/NMM fluorescent probe (Figure 11A) [104]. The oligonucleotide AGRO100 can be induced by K^+^ to fold into a G-quadruplex structure and bind to NMM to produce strong fluorescence. When the target Pb^2+^ is present, it acts as a competing ion to replace K^+^ and induces reassembly of the G-quadruplex. The fluorescence intensity is reduced due to the weakened interaction of the Pb^2+^-induced G-quadruplex structure with NMM. The T base in the AGRO100 probe can bind to Hg^2+^ via T-Hg^2+^-T interaction, which induces the transformation of the G-quadruplex structure into a hairpin-like structure, reducing the interaction with NMM and leading to a decrease in fluorescence intensity. By achieving an LOD as low as 5.0 nM for Pb^2+^ and 18.6 nM for Hg^2+^, this biosensor can provide precise and reliable results when analyzing actual lake samples.

In addition to the aforementioned metal ions, G-quadruplex sensors exhibit the capability to monitor K^+^ ions in lysosomes in human cells. In 2020, Chen et al. created two novel DNA sensors for simultaneous fluorescence analysis of pH and K^+^ in the lysosomal cavity (Figure 11B) [105]. These two sensors are based on two upconversion nanoparticles (UCNPs) with different emission wavelengths as emitters and AuNPs as quenchers. When the target H^+^ and K^+^ are present, the H^+^-induced DNA triplex and K^+^-induced G-quadruplex are formed, respectively, causing the quencher AuNP to become close to UCNP. The energy transferred between the two nanoparticles leads to the quenching of UCNP fluorescence. These sensors can respond to pH and K^+^ in vitro and in vivo with high selectivity.

**Figure 12:**
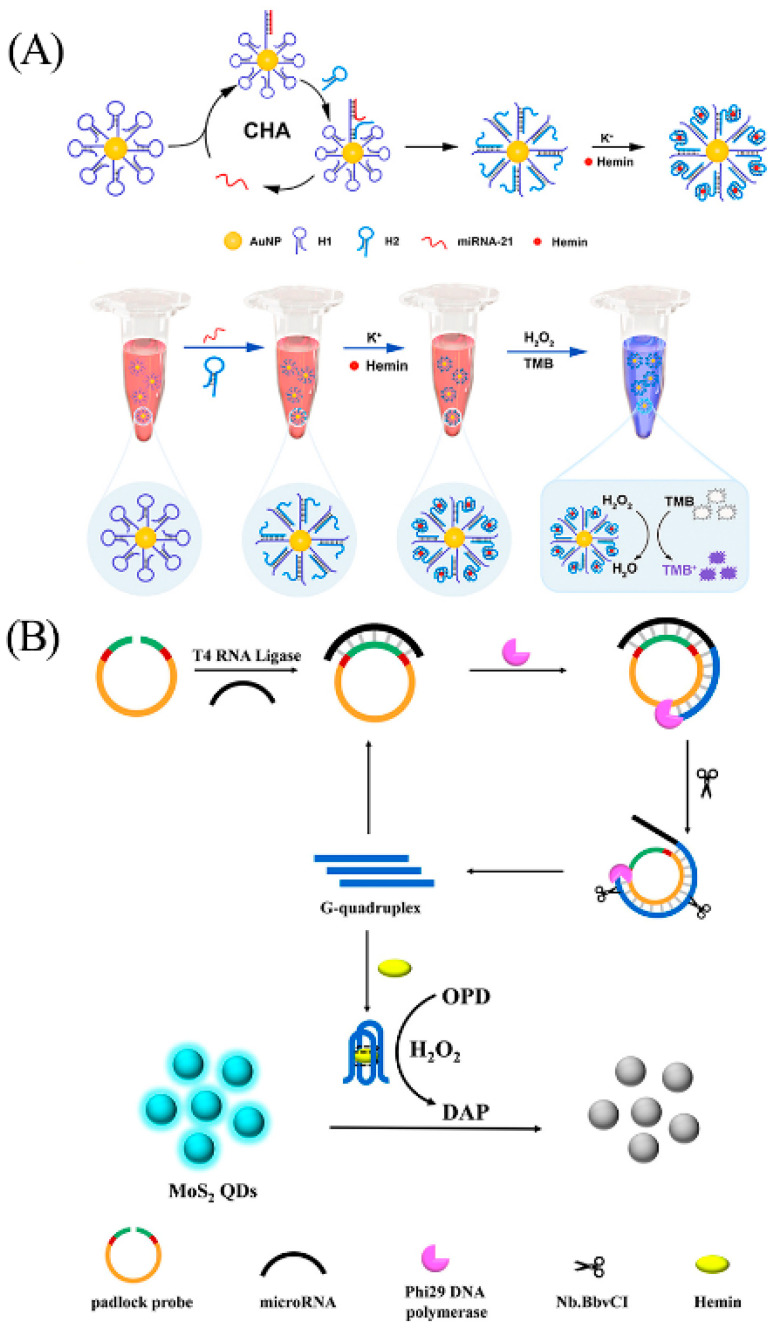
(**A**) Principle and design of colorimetric detection of microRNA. Reprinted with permission from [107]. Copyright (2023) Elsevier. (**B**) Principle and design of detection of microRNA via IFE of MoS_2_ QDs. Reprinted with permission from [110]. Copyright (2020) American Chemical Society.

The utilization of biosensors based on G-quadruplex/hemin holds great potential for detecting miRNA. Colorimetry, a technique dependent on the analysis of wavelength and intensity of light, exploits the optical attributes of the sensing element to produce the output signal for effective detection [106]. Yang et al. combined catalytic hairpin assembly (CHA) and enzyme-catalyzed chromogenic reactions to develop a G-quadruplex/hemin colorimetric sensing strategy for sensitive analysis of miRNAs (Figure 12A) [107]. First, the authors assembled many hairpin oligonucleotides 1 (H1) on AuNP. Upon interaction with the target miRNA, H1 opens and hybridizes with miRNA and hairpin oligonucleotides 2 (H2), ultimately exposing the G-rich sequences. In the presence of K^+^, G-rich sequences bound to hemin form G-quadruplex/hemin to catalyze the chromogenic oxidation of 3,3′,5,5′-tetramethylbenzidine (TMB) and producing color changes. As an example, the method exhibits remarkable specificity and high sensitivity for miRNA-21, with a detection limit of 90.3 fM. In addition, the sensor platform was successfully employed to detect miRNA-21 in serum, providing a promising tool for the early diagnosis of cancer. Colorimetric analysis, renowned for its simplicity, cost-effectiveness, and visible detection signal, is widely used. However, it is worth mentioning that this method can be susceptible to environmental influences [108].

The inner filter effect (IFE) refers to the absorption of excitation light or emission light (or both) by the sample matrix, resulting in the decrease in fluorescence intensity of the fluorophore [109]. The fluorescence determination method based on IFE effect has high requirements for the extinction coefficients of absorbers. In 2020, based on the nicking-enhanced RCA signal amplification method, Ge et al. successfully developed a G-quadruplex/hemin sensor based on the IFE quenching effect of molybdenum disulfide (MoS_2_) (Figure 12B) [110]. The padlock probe designed by the authors specifically recognizes microRNA let-6a, then undergoes a ligation and RCA reaction, and finally produces many G quadruplexes facilitated by the action of the nicking endonuclease. In the presence of hemin, the G-quadruplex/hemin forms and catalyzes the oxidation of o-phenylenediamine (OPD) to form 2, 3-diaminophenazine (DAP), which decreases the fluorescence intensity of MoS_2_ QDs. The detection limit of this sensor for miRNA let-6a is 7.4 fM, and it also has an impressive detection effect for miRNA let-6a in serum.

**Figure 13:**
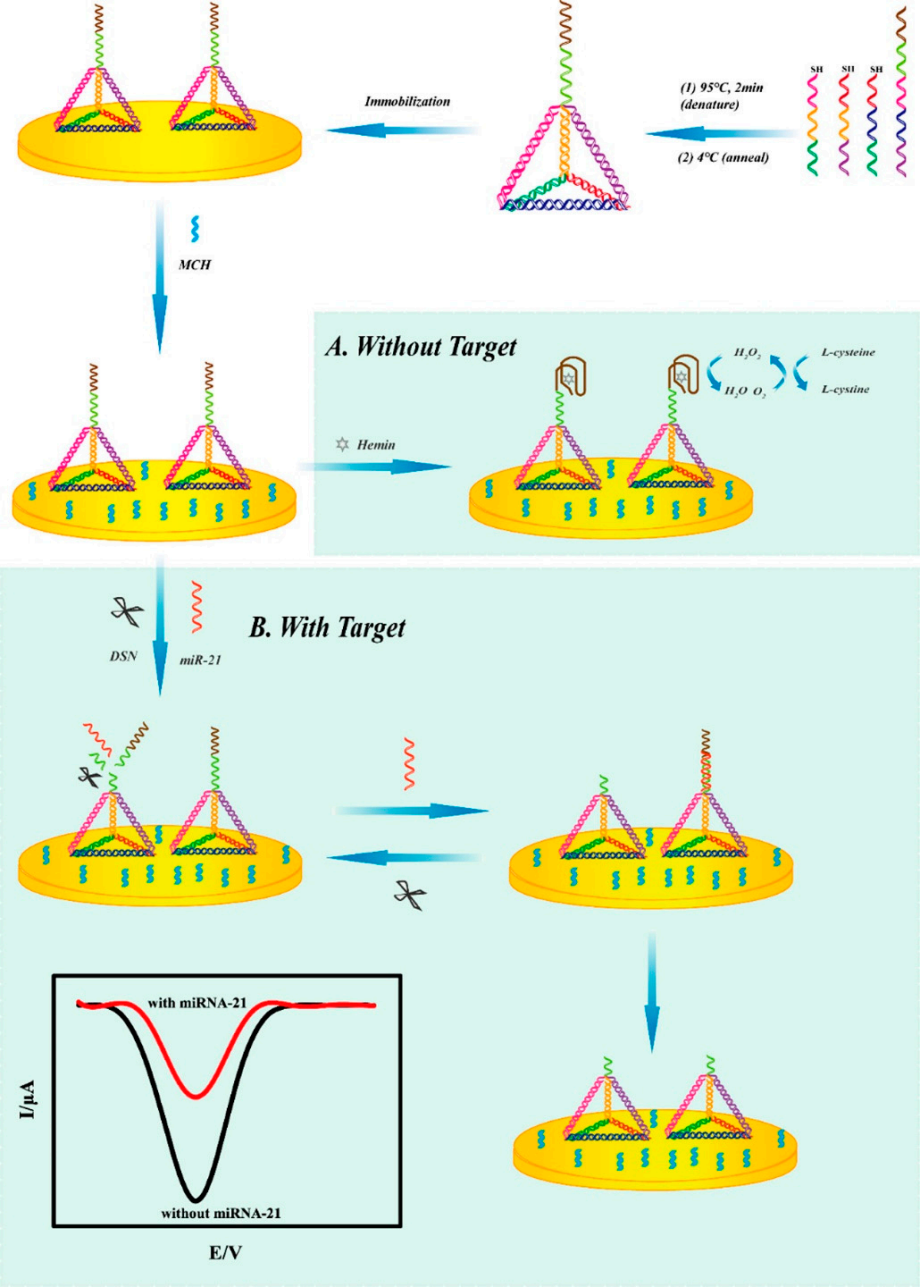
Illustration of the operation principle and design of the electrochemical detection system without the target microRNA (**A**) and with the target microRNA (**B**). Reprinted with permission from [112]. Copyright (2019) American Chemical Society.

In addition, electrochemical biosensors offer distinct advantages in terms of cost, efficiency, and precision, making them extensively used in miRNA detection [111]. In 2019, Lu et al. constructed a novel electrochemical biosensor based on G-quadruplex/hemin and DNA tetrahedral structures for the rapid detection of miRNA-21 in serum (Figure 13) [112]. In the absence of miRNA-21, L-cysteine is oxidized to produce H_2_O_2_; then, G-quadruplex/hemin catalyzes the oxidation of H_2_O_2_, and the system generates a stable electrical signal through this process. In the presence of target, the miRNA hybridizes with the G-rich sequence and forms a DNA–RNA duplex. The duplex-specific nuclease (DSN) in solution then specifically cleaves the DNA strand in the heterologous duplex. This results in disruption of the G-quadruplex/hemin complex and its failure in catalyzing the reaction, consequently leading to a diminished electrical signal. Under optimal conditions, the sensor exhibits a linear range of 0.1 fM to 0.1 pM and a detection limit as low as 0.04 fM.

**Figure 14:**
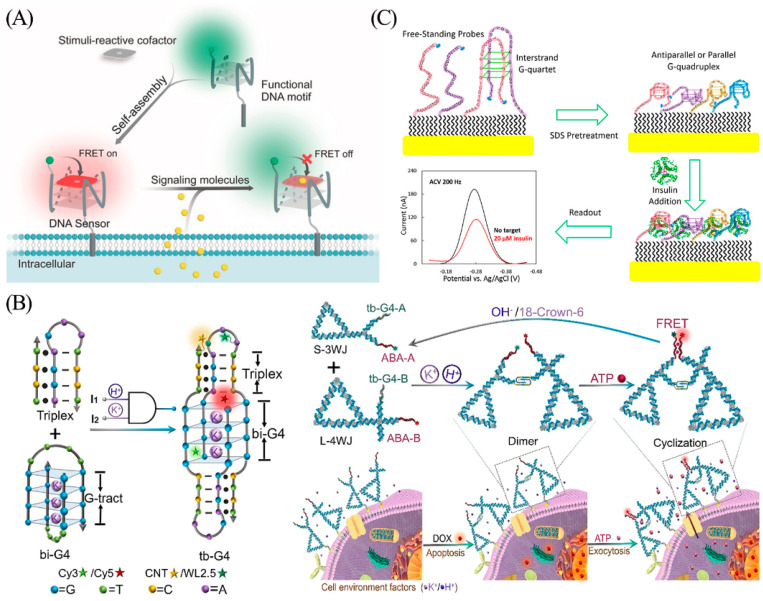
(**A**) Schematic illustration of the G-quadruplex and synthetic cofactors for monitoring of SO_2_ derivatives and NO in the cellular membrane microenvironment. Reprinted with permission from [115]. Copyright (2019) Wiley-VCH GmbH. (**B**) Schematic illustration for tb-G4s logic-gated nanoplatform and ATP aptasensor for real-time monitoring of ATP in cells. Reprinted with permission from [117]. Copyright (2021) Wiley-VCH GmbH. (**C**) Schematic illustration of a binding-induced steric hindrance electrochemical strategy for monitoring of insulin. Reprinted with permission from [120]. Copyright (2019) American Chemical Society.

Molecules that interact with normal physiological and biochemical processes in the body and exhibit some physiological activities are referred to as endogenous substances in the general definition [113]. Nitric oxide (NO), carbon monoxide (CO), and hydrogen sulfide (H_2_S) are endogenous gasotransmitters that play important roles in cellular communication and signaling in living organisms [114]. Nie’s team integrated synthetic cofactors with G-quadruplexes to construct a DNA-based sensor to achieve live cell imaging analysis of SO_2_ derivatives and NO signaling molecules within the intricate microenvironment of the cellular membrane. (Figure 14A) [115]. When a cell secretes signal molecules (such as SO_2_ derivatives or NO), recognition of the signal molecule by the responsive cofactor leads to a change in its fluorescence properties and turns off the fluorescence signal by FRET. This enables ratiometric fluorescence imaging analysis of gas signaling molecules in the plasma membrane microenvironment.

ATP (adenosine 5′-triphosphate) is another pivotal endogenous signaling molecule involved in immunity and inflammation, wherein apoptotic cells release ATP to trigger swift clearance by phagocytes [116]. To achieve real-time detection of apoptosis, Li et al. designed a triple-helix-enhanced G-quadruplex (tb-G4s) with H^+^ and K^+^ synergistic response for real-time monitoring of extracellular secretion of ATP (Figure 14B) [117]. A pH-induced triplex sequence in the K^+^-induced bimolecular G-quadruplex (bi-G4) stem-loop forms a triplex-enhanced G-quadruplex (tb-G4) topology that undergoes heterodimer folding only in the presence of both H^+^ and K^+^. Based on this, the authors used DNA nanotriangles as a nucleic acid framework to achieve heterologous binding of tb-G4 by attaching three- and four-armed structures with tb-G4 and ATP aptamers at the pivot points, and then assembled proximity-induced dimeric nanostructures to promote specific binding of ATP and induce FRET effects. Based on the FRET signal generated by the system, the ATP released by the cells can be accurately quantified. Here, the G-quadruplex plays a dual role by guiding the functional nucleic acid assembly, as well as serving as a logical gating for proximity sensing.

Insulin, a vital protein hormone, is secreted by pancreatic beta cells. Secretion of insulin is stimulated by endogenous or exogenous substances such as glucose, lactose, ribose, arginine, and glucagon [118]. The type 2 diabetes phenotype arises from a combination of insulin deficiency and insulin resistance, highlighting the importance of insulin testing in the diagnosis, treatment, and prognosis of diabetes [119]. Wu et al. used redox-labeled modified G-quadruplexes to construct an electrochemical DNA sensor for real-time insulin monitoring (Figure 14C) [120]. The interstrand G-quadruplexes are disrupted by pretreatment with 10% sodium dodecyl sulfate (SDS). After binding to the insulin target, the binding-induced steric hindrance created by the complex quantitatively reduces the electron transfer efficiency of the redox label (MB), resulting in a decreased electrical signal. The sensor is capable of rapid, specific, and quantitative analysis of insulin with a detection limit of 20 nM.

## 3. Summary and Prospects

In general, DNA has the advantages of structural programmability, response predictability, and high thermal stability, making it an excellent foundational element in the construction of biosensors. As disease surveillance becomes increasingly important, the adoption of biosensors is expected to grow in the future. The DNA-based sensing approach discussed in this review is characterized by superior sensitivity, specificity, and analysis speed. However, there is still much room for improvement. In this section, we discuss the challenges and opportunities associated with DNA sensing.

First, DNA is easily polluted and degraded, and its storage conditions are strict, limiting its widespread application. To address these issues, researchers are exploring site-specific modifications to the DNA and incorporating DNA nanostructures into sensors, thereby alleviating degradation to a certain extent. These DNA nanomaterials can be broadly classified into two categories: self-assembled materials composed of pure DNA, and nanomaterials synthesized by combining the DNA to other materials with certain properties.

In the self-assembled DNA materials, a key approach involves the creation of multi-stranded nucleic acid structures through sequence complementarity. This strategy imparts increased resistance to enzymatic cleavage, enhancing the stability of structures such as DNA tetrahedrons. Remarkably, this enhances their anti-degradation capability while preserving biocompatibility. Moreover, by making full use of the programmable characteristics of DNA nanocomplex, researchers can synthesize DNA materials that perfectly match the size and shape of analytes. Examples include DNA bricks and DNA origami, which represent a milestone in DNA nanotechnology. Through sequence design, DNA brick and DNA origami can be designed into structures of various sizes and shapes. There is no doubt that these precisely controllable and programmable tools are excellent detection elements. For example, Raveendran et al. designed an origami with a cavity for single-molecule detection. Upon analyte interaction with the well-designed cavity part, the characteristic ion current peak is produced. Experiments have shown that the rigidity of the origami structure reduces false positive signals, and the cavity structure also increases signal sensitivity [121].

Expanding beyond the field of pure-DNA based nanostructures, researchers have successfully integrated inorganic materials such as metal nanoparticles. The nano-flare designed by Mirkin’s research group is a case in point, employing the fluorescence-quenching properties of gold nanoparticles to sensitively detect mRNA in cells [122]. The spherical nucleic acids developed by Mirkin’s research group are centered on metal nanoparticles, and nucleic acids are arranged with high density on the metal surface. This structure enhances the resistance to nuclease degradation, and the high-density nucleic acids on the surface also improve reaction rates [123,124]. There are also studies using carbon nanotubes [125], graphene [126], upconversion materials [127,128], or micelles [129,130] to engineer DNA nanostructures. This strategic incorporation of materials with diverse properties has greatly broadened the application scenarios of DNA sensing.

The second challenge lies in the notable difference between laboratory samples and actual samples. Most reported sensing elements are screened under well-controlled conditions and perform well in relatively simple systems, exhibiting binding constants to targets in the micro- to picomolar range. However, these proof-of-concept studies often focus on single components of interest, which cannot perfectly simulate the actual complex biological system. Even minor variations in clinical samples or small changes in detection conditions, such as body fluid pH, ionic strength, and temperature, may significantly impact the sensor’s performance. To tackle this challenge, ongoing research is dedicated to the continuous evolution and improvement of functional nucleic acids, such as aptamers, with increasingly diverse and enhanced functions. Moreover, researchers are actively screening specific sensing elements directly from complex clinical samples. These endeavors hold the potential to apply biosensors into complex systems.

Additionally, we believe that DNA-based biosensors hold great potential to address this challenge with their excellent data storage capabilities and computational power. In recent years, using DNA to construct molecular computing machines and to solve practical problems in different fields has become an international frontier research hotspot. With continuing advances in biotechnology, such as high-throughput sequencing, and the integration of cutting-edge artificial intelligence technologies such as machine learning, the engineering of DNA systems enables simultaneous analysis of multiple analytes, rapid computing, and accurate conclusion output. For instance, Han’s group was committed to manufacturing nucleic acid devices with artificial intelligence to classify lung cancer individuals from healthy individuals [131]. Progress in DNA biosensing will greatly contribute to the development of personalized medicine, facilitating personalized disease prevention, diagnosis, and treatment. In the future, people will have greater control over their own health data and participate more in the exchange of research data [132].

Third, the detection cost of using DNA sensors is relatively high at the current stage. While DNA itself offers cost advantages, with DNA synthesis companies in China able to synthesize 100 nmol of unmodified 30-base single-stranded DNA for only USD 30, the overall expense of DNA sensing is influenced by the requirement of large, sophisticated instruments and skilled operators. This restricts accessibility for families and individuals. To address this challenge, an important focus in the development of DNA sensing technology is the creation of simple, rapid, cost-effective, and convenient detection methods for POCT [133,134,135].

As technology advances, the evolution of microfluidic electronic chips, microelectromechanical systems, and soft wearable materials [25] will make DNA sensing more practical and affordable. Zhang and colleagues developed Drop-BS, a droplet-based microfluidic technology for building single-cell bisulfite sequencing libraries, thus facilitating DNA methylation analysis [136]. Drop-BS uses droplet microfluidics to enable extremely high throughput and prepare up to 10,000 single-cell bisulfite sequencing libraries in just two days. This provides a promising solution for realizing the high-throughput analysis of methylation. Wang et al. immobilized DNA tetrahedra with aptamer probes on a liquid-gated graphene field-effect transistor to detect nasopharyngeal samples from 33 COVID-19 patients within four minutes without RNA extraction or nucleic acid amplification [126]. These progressions can enable on-site testing in remote areas or in emergency situations, reducing reliance on specialized equipment and personnel.

The methods used in DNA sensing are continuously evolving, and the most recent technologies hold promise for the future of DNA sensing. As mentioned above, one trend focuses on accurate analysis of complex samples with DNA’s excellent data storage ability and computing power, while another trend is the development of DNA sensing technology for on-site detection. No matter which direction DNA analysis develops, its ultimate destination remains constant—to become a critical tool in various fields by virtue of its unique advantages.

## Data Availability

Not applicable.

## References

[1] Vargas E., Zhang F., Ben Hassine A., Ruiz-Valdepeñas Montiel V., Mundaca-Uribe R., Nandhakumar P., He P., Guo Z., Zhou Z., Fang R.H. (2022). Using Cell Membranes as Recognition Layers to Construct Ultrasensitive and Selective Bioelectronic Affinity Sensors. J. Am. Chem. Soc..

[2] Walgama C., Nerimetla R., Materer N.F., Schildkraut D., Elman J.F., Krishnan S. (2015). A simple construction of electrochemical liver microsomal bioreactor for rapid drug metabolism and inhibition assays. Anal. Chem..

[3] Huang Z., Li J., Zhong H., Tian B. (2022). Nucleic acid amplification strategies for volume-amplified magnetic nanoparticle detection assay. Front. Bioeng. Biotechnol..

[4] Pohanka M. (2018). Overview of piezoelectric biosensors, immunosensors and DNA sensors and their applications. Materials.

[5] Ellington A.D., Szostak J.W. (1990). In vitro selection of RNA molecules that bind specific ligands. Nature.

[6] Tuerk C., Gold L. (1990). Systematic evolution of ligands by exponential enrichment: RNA ligands to bacteriophage T4 DNA polymerase. Science.

[7] Stoltenburg R., Reinemann C., Strehlitz B. (2007). SELEX—A (r)evolutionary method to generate high-affinity nucleic acid ligands. Biomol. Eng..

[8] Shangguan D., Li Y., Tang Z., Cao Z.C., Chen H.W., Mallikaratchy P., Sefah K., Yang C.J., Tan W. (2006). Aptamers evolved from live cells as effective molecular probes for cancer study. Proc. Natl. Acad. Sci. USA.

[9] Niu W., Jiang N., Hu Y. (2007). Detection of proteins based on amino acid sequences by multiple aptamers against tripeptides. Anal. Biochem..

[10] Farokhzad O.C., Cheng J., Teply B.A., Sherifi I., Jon S., Kantoff P.W., Richie J.P., Langer R. (2006). Targeted nanoparticle-aptamer bioconjugates for cancer chemotherapy in vivo. Proc. Natl. Acad. Sci. USA.

[11] Zhao W., Chiuman W., Lam J.C., McManus S.A., Chen W., Cui Y., Pelton R., Brook M.A., Li Y. (2008). DNA aptamer folding on gold nanoparticles: From colloid chemistry to biosensors. J. Am. Chem. Soc..

[12] Qu H., Csordas A.T., Wang J., Oh S.S., Eisenstein M.S., Soh H.T. (2016). Rapid and label-free strategy to isolate aptamers for metal ions. ACS Nano.

[13] Fan C., Plaxco K.W., Heeger A.J. (2003). Electrochemical interrogation of conformational changes as a reagentless method for the sequence-specific detection of DNA. Proc. Natl. Acad. Sci. USA.

[14] Liu G., Wan Y., Gau V., Zhang J., Wang L., Song S., Fan C. (2008). An enzyme-based E-DNA sensor for sequence-specific detection of femtomolar DNA targets. J. Am. Chem. Soc..

[15] Lin M., Wang J., Zhou G., Wang J., Wu N., Lu J., Gao J., Chen X., Shi J., Zuo X. (2015). Programmable engineering of a biosensing interface with tetrahedral DNA nanostructures for ultrasensitive DNA detection. Angew. Chem. Int. Ed..

[16] Zhao L., Qi X., Yan X., Huang Y., Liang X., Zhang L., Wang S., Tan W. (2019). Engineering aptamer with enhanced affinity by triple helix-based terminal fixation. J. Am. Chem. Soc..

[17] Feagin T.A., Maganzini N., Soh H.T. (2018). Strategies for creating structure-switching aptamers. ACS Sens..

[18] Victorious A., Zhang Z., Chang D., Maclachlan R., Pandey R., Xia J., Gu J., Hoare T., Soleymani L., Li Y. (2022). A DNA Barcode-Based Aptasensor Enables Rapid Testing of Porcine Epidemic Diarrhea Viruses in Swine Saliva Using Electrochemical Readout. Angew. Chem..

[19] Siavash Moakhar R., Mahimkar R., Khorrami Jahromi A., Mahshid S.S., del Real Mata C., Lu Y., Vasquez Camargo F., Dixon B., Gilleard J., J Da Silva A. (2023). Aptamer-Based Electrochemical Microfluidic Biosensor for the Detection of *Cryptosporidium parvum*. ACS Sens..

[20] Hou H., Jin Y., Wei H., Ji W., Xue Y., Hu J., Zhang M., Jiang Y., Mao L. (2020). A generalizable and noncovalent strategy for interfacing aptamers with a microelectrode for the selective sensing of neurotransmitters in vivo. Angew. Chem. Int. Ed..

[21] Bošković F., Zhu J., Tivony R., Ohmann A., Chen K., Alawami M.F., Đorđević M., Ermann N., Pereira-Dias J., Fairhead M. (2023). Simultaneous identification of viruses and viral variants with programmable DNA nanobait. Nat. Nanotechnol..

[22] Wienken C.J., Baaske P., Rothbauer U., Braun D., Duhr S. (2010). Protein-binding assays in biological liquids using microscale thermophoresis. Nat. Commun..

[23] Li Y., Deng J., Han Z., Liu C., Tian F., Xu R., Han D., Zhang S., Sun J. (2021). Molecular Identification of Tumor-Derived Extracellular Vesicles Using Thermophoresis-Mediated DNA Computation. J. Am. Chem. Soc..

[24] Dirks R.M., Pierce N.A. (2004). Triggered amplification by hybridization chain reaction. Proc. Natl. Acad. Sci. USA.

[25] An J.E., Kim K.H., Park S.J., Seo S.E., Kim J., Ha S., Bae J., Kwon O.S. (2022). Wearable cortisol aptasensor for simple and rapid real-time monitoring. ACS Sens..

[26] Willner I., Shlyahovsky B., Zayats M., Willner B. (2008). DNAzymes for sensing, nanobiotechnology and logic gate applications. Chem. Soc. Rev..

[27] Liu J., Cao Z., Lu Y. (2009). Functional nucleic acid sensors. Chem. Rev..

[28] Yang X., Fang C., Mei H., Chang T., Cao Z., Shangguan D. (2011). Characterization of G-Quadruplex/Hemin Peroxidase: Substrate Specificity and Inactivation Kinetics. Chem. Eur. J..

[29] Xiong J., Dong C., Zhang J., Fang X., Ni J., Gan H., Li J., Song C. (2022). DNA walker-powered ratiometric SERS cytosensor of circulating tumor cells with single-cell sensitivity. Biosens. Bioelectron..

[30] Li D., Zhao T., Chen J., Shi J., Wang J., Yin Y., Chen S., Xu S., Luo X. (2022). Spatiotemporally controlled ultrasensitive molecular imaging using a DNA computation-mediated DNAzyme platform. Anal. Chem..

[31] Sfrazzetto G.T., Satriano C., Tomaselli G.A., Rizzarelli E. (2016). Synthetic fluorescent probes to map metallostasis and intracellular fate of zinc and copper. Coord. Chem. Rev..

[32] Hawtrey T., New E.J. (2023). Molecular probes for fluorescent sensing of metal ions in non-mammalian organisms. Curr. Opin. Chem. Biol..

[33] Chang C.J. (2015). Searching for harmony in transition-metal signaling. Nat. Chem. Biol..

[34] Yi D., Zhao H., Zhao J., Li L. (2022). Modular Engineering of DNAzyme-Based Sensors for Spatioselective Imaging of Metal Ions in Mitochondria. J. Am. Chem. Soc..

[35] Etheridge W., Brossard F., Zheng S., Moench S., Pavagada S., Owens R.M., Fruk L. (2023). Activity-enhanced DNAzyme for design of label-free copper (ii) biosensor. Nanoscale.

[36] Liu J., Brown A.K., Meng X., Cropek D.M., Istok J.D., Watson D.B., Lu Y. (2007). A catalytic beacon sensor for uranium with parts-per-trillion sensitivity and millionfold selectivity. Proc. Natl. Acad. Sci. USA.

[37] Wu P., Hwang K., Lan T., Lu Y. (2013). A DNAzyme-gold nanoparticle probe for uranyl ion in living cells. J. Am. Chem. Soc..

[38] He X., Zhou X., Liu Y., Wang X. (2020). Ultrasensitive, recyclable and portable microfluidic surface-enhanced raman scattering (SERS) biosensor for uranyl ions detection. Sens. Actuators B Chem..

[39] Wightman B., Ha I., Ruvkun G. (1993). Posttranscriptional regulation of the heterochronic gene lin-14 by lin-4 mediates temporal pattern formation in *C. elegans*. Cell.

[40] Treiber T., Treiber N., Meister G. (2019). Regulation of microRNA biogenesis and its crosstalk with other cellular pathways. Nat. Rev. Mol. Cell Biol..

[41] Small E.M., Olson E.N. (2011). Pervasive roles of microRNAs in cardiovascular biology. Nature.

[42] Yokoi A., Matsuzaki J., Yamamoto Y., Yoneoka Y., Takahashi K., Shimizu H., Uehara T., Ishikawa M., Ikeda S.-I., Sonoda T. (2018). Integrated extracellular microRNA profiling for ovarian cancer screening. Nat. Commun..

[43] Garo L.P., Ajay A.K., Fujiwara M., Gabriely G., Raheja R., Kuhn C., Kenyon B., Skillin N., Kadowaki-Saga R., Saxena S. (2021). MicroRNA-146a limits tumorigenic inflammation in colorectal cancer. Nat. Commun..

[44] Dhawan A., Scott J.G., Harris A.L., Buffa F.M. (2018). Pan-cancer characterisation of microRNA across cancer hallmarks reveals microRNA-mediated downregulation of tumour suppressors. Nat. Commun..

[45] Rastegar-Moghaddam S.H., Ebrahimzadeh-Bideskan A., Shahba S., Malvandi A.M., Mohammadipour A. (2023). Roles of the miR-155 in neuroinflammation and neurological disorders: A potent biological and therapeutic target. Cell. Mol. Neurobiol..

[46] Rastegar-Moghaddam S.H., Ebrahimzadeh-Bideskan A., Shahba S., Malvandi A.M., Mohammadipour A. (2022). MicroRNA-22: A novel and potent biological therapeutics in neurological disorders. Mol. Neurobiol..

[47] Pritchard C.C., Cheng H.H., Tewari M. (2012). MicroRNA profiling: Approaches and considerations. Nat. Rev. Genet..

[48] Hu C., Zhang L., Yang Z., Song Z., Zhang Q., He Y. (2021). Graphene oxide-based qRT-PCR assay enables the sensitive and specific detection of miRNAs for the screening of ovarian cancer. Anal. Chim. Acta.

[49] Wei J., Wang H., Wu Q., Gong X., Ma K., Liu X., Wang F. (2020). A smart, autocatalytic, DNAzyme biocircuit for in vivo, amplified, microRNA imaging. Angew. Chem..

[50] Wang J., Yu S., Wu Q., Gong X., He S., Shang J., Liu X., Wang F. (2021). A self-catabolic multifunctional DNAzyme nanosponge for programmable drug delivery and efficient gene silencing. Angew. Chem. Int. Ed..

[51] Gao Y., Zhang S., Wu C., Li Q., Shen Z., Lu Y., Wu Z.-S. (2021). Self-protected DNAzyme walker with a circular bulging DNA shield for amplified imaging of miRNAs in living cells and mice. ACS Nano.

[52] Yin Y., Chen G., Gong L., Ge K., Pan W., Li N., Machuki J., Yu Y., Geng D., Dong H. (2020). DNAzyme-powered three-dimensional DNA walker nanoprobe for detection amyloid β-peptide oligomer in living cells and in vivo. Anal. Chem..

[53] Zhao J., He C., Long Y., Lei J., Liu H., Hou J., Hou C., Huo D. (2023). 3D DNAzyme walker based electrochemical biosensor for attomolar level microRNA-155 detection. Anal. Chim. Acta.

[54] Chorti P., Kazi A.P., Wiederoder M., Christodouleas D.C. (2021). High-Throughput Flow-Through Direct Immunoassays for Targeted Bacteria Detection. Anal. Chem..

[55] Guo Q., Han J.-J., Shan S., Liu D.-F., Wu S.-S., Xiong Y.-H., Lai W.-H. (2016). DNA-based hybridization chain reaction and biotin–streptavidin signal amplification for sensitive detection of Escherichia coli O157: H7 through ELISA. Biosens. Bioelectron..

[56] Yamashige R., Kimoto M., Okumura R., Hirao I. (2018). Visual detection of amplified DNA by polymerase chain reaction using a genetic alphabet expansion system. J. Am. Chem. Soc..

[57] Ma X., Ding W., Wang C., Wu H., Tian X., Lyu M., Wang S. (2021). DNAzyme biosensors for the detection of pathogenic bacteria. Sens. Actuators B Chem..

[58] Xing G., Zhang W., Li N., Pu Q., Lin J.-M. (2022). Recent progress on microfluidic biosensors for rapid detection of pathogenic bacteria. Chin. Chem. Lett..

[59] Tram K., Kanda P., Salena B.J., Huan S., Li Y. (2014). Translating bacterial detection by DNAzymes into a litmus test. Angew. Chem..

[60] Ali M.M., Wolfe M., Tram K., Gu J., Filipe C.D., Li Y., Brennan J.D. (2019). A DNAzyme-based colorimetric paper sensor for Helicobacter pylori. Angew. Chem..

[61] Pandey R., Chang D., Smieja M., Hoare T., Li Y., Soleymani L. (2021). Integrating programmable DNAzymes with electrical readout for rapid and culture-free bacterial detection using a handheld platform. Nat. Chem..

[62] Gehring K., Leroy J.-L., Guéron M. (1993). A tetrameric DNA structure with protonated cytosine-cytosine base pairs. Nature.

[63] Liu D., Balasubramanian S. (2003). A proton-fuelled DNA nanomachine. Angew. Chem. Int. Ed..

[64] Phan A.T., Guéron M., Leroy J.-L. (2000). The solution structure and internal motions of a fragment of the cytidine-rich strand of the human telomere. J. Mol. Biol..

[65] Zeraati M., Langley D.B., Schofield P., Moye A.L., Rouet R., Hughes W.E., Bryan T.M., Dinger M.E., Christ D. (2018). I-motif DNA structures are formed in the nuclei of human cells. Nat. Chem..

[66] Nonin-Lecomte S., Leroy J.L. (2001). Structure of a C-rich strand fragment of the human centromeric satellite III: A pH-dependent intercalation topology. J. Mol. Biol..

[67] Brooks T.A., Kendrick S., Hurley L. (2010). Making sense of G-quadruplex and i-motif functions in oncogene promoters. FEBS J..

[68] Kaiser C.E., Van Ert N.A., Agrawal P., Chawla R., Yang D., Hurley L.H. (2017). Insight into the complexity of the i-motif and G-quadruplex DNA structures formed in the KRAS promoter and subsequent drug-induced gene repression. J. Am. Chem. Soc..

[69] Justus C.R., Dong L., Yang L.V. (2013). Acidic tumor microenvironment and pH-sensing G protein-coupled receptors. Front. Physiol..

[70] Zeng S., Liu D., Li C., Yu F., Fan L., Lei C., Huang Y., Nie Z., Yao S. (2018). Cell-surface-anchored ratiometric DNA tweezer for real-time monitoring of extracellular and apoplastic pH. Anal. Chem..

[71] Yang H., Chen J., Liang Y., Zhang Y., Yin W., Xu Y., Liu S.-Y., Dai Z., Zou X. (2021). A MOF-Shell-Confined I-Motif-Based pH Probe (MOFC-i) Strategy for Sensitive and Dynamic Imaging of Cell Surface pH. ACS Appl. Mater. Interfaces.

[72] Bus T., Traeger A., Schubert U.S. (2018). The great escape: How cationic polyplexes overcome the endosomal barrier. J. Mater. Chem. B.

[73] Banushi B., Joseph S.R., Lum B., Lee J.J., Simpson F. (2023). Endocytosis in cancer and cancer therapy. Nat. Rev. Cancer.

[74] Gao Y., Chen X., Tian T., Zhang T., Gao S., Zhang X., Yao Y., Lin Y., Cai X. (2022). A lysosome-activated tetrahedral Nanobox for encapsulated siRNA delivery. Adv. Mater..

[75] He S., Liu M., Yin F., Liu J., Ge Z., Li F., Li M., Shi J., Wang L., Mao X. (2021). Programming folding cooperativity of the dimeric i-motif with DNA frameworks for sensing small pH variations. Chem. Commun..

[76] Nesterova I.V., Nesterov E.E. (2014). Rational design of highly responsive pH sensors based on DNA i-motif. J. Am. Chem. Soc..

[77] Yue X., Qiao Y., Gu D., Qi R., Zhao H., Yin Y., Zhao W., Xi R., Meng M. (2021). DNA-based pH nanosensor with adjustable FRET responses to track lysosomes and pH fluctuations. Anal. Chem..

[78] Liu J., Jing X., Liu M., Li F., Li M., Li Q., Shi J., Li J., Wang L., Mao X. (2022). Mechano-fluorescence actuation in single synaptic vesicles with a DNA framework nanomachine. Sci. Robot..

[79] Ghosal G., Muniyappa K. (2006). Hoogsteen base-pairing revisited: Resolving a role in normal biological processes and human diseases. Biochem. Biophys. Res. Commun..

[80] Rhodes D., Lipps H.J. (2015). G-quadruplexes and their regulatory roles in biology. Nucleic Acids Res..

[81] Georgiades S., Abd Karim N., Suntharalingam K., Vilar R. (2009). Interaction of Metal Complexes with G-Quadruplex DNA. Angew. Chem. Int. Ed..

[82] Roxo C., Kotkowiak W., Pasternak A. (2019). G-quadruplex-forming aptamers-characteristics, applications, and perspectives. Molecules.

[83] Bhattacharyya D., Mirihana Arachchilage G., Basu S. (2016). Metal cations in G-quadruplex folding and stability. Front. Chem..

[84] Largy E., Mergny J.L., Gabelica V., Sigel A., Sigel H., Sigel R. (2016). Role of alkali metal ions in G-quadruplex nucleic acid structure and stability. The Alkali Metal Ions: Their Role for Life.

[85] Xu J., Jiang R., Feng Y., Liu Z., Huang J., Ma C., Wang K. (2022). Functional nucleic acid-based fluorescent probes for metal ion detection. Coord. Chem. Rev..

[86] Pathak P., Yao W., Hook K.D., Vik R., Winnerdy F.R., Brown J.Q., Gibb B.C., Pursell Z.F., Phan A.T., Jayawickramarajah J. (2019). Bright G-quadruplex nanostructures functionalized with porphyrin lanterns. J. Am. Chem. Soc..

[87] Yang H., Zhou Y., Liu J. (2020). G-quadruplex DNA for construction of biosensors. Trends Anal. Chem..

[88] Yett A., Lin L.Y., Beseiso D., Miao J., Yatsunyk L.A. (2019). N-methyl mesoporphyrin IX as a highly selective light-up probe for G-quadruplex DNA. J. Porphyr. Phthalocyanines.

[89] Bhasikuttan A.C., Mohanty J. (2015). Targeting G-quadruplex structures with extrinsic fluorogenic dyes: Promising fluorescence sensors. Chem. Commun..

[90] Bhasikuttan A.C., Mohanty J., Pal H. (2007). Interaction of malachite green with guanine-rich single-stranded DNA: Preferential binding to a G-quadruplex. Angew. Chem. Int. Ed..

[91] Travascio P., Li Y., Sen D. (1998). DNA-enhanced peroxidase activity of a DNA aptamer-hemin complex. Chem. Biol..

[92] Li J., Wu H., Yan Y., Yuan T., Shu Y., Gao X., Zhang L., Li S., Ding S., Cheng W. (2021). Zippered G-quadruplex/hemin DNAzyme: Exceptional catalyst for universal bioanalytical applications. Nucleic Acids Res..

[93] Yum J.H., Park S., Sugiyama H. (2019). G-quadruplexes as versatile scaffolds for catalysis. Org. Biomol. Chem..

[94] Alizadeh N., Salimi A., Hallaj R., Seitz H., Stahl F., Walter J.G. (2020). Hemin/G-quadruplex horseradish peroxidase-mimicking DNAzyme: Principle and biosensing application. Catalytically Active Nucleic Acids.

[95] Mehta N., Benzerara K., Kocar B.D., Chapon V. (2019). Sequestration of radionuclides radium-226 and strontium-90 by cyanobacteria forming intracellular calcium carbonates. Environ. Sci. Technol..

[96] Newcombe H. (1957). Magnitude of biological hazard from strontium-90. Science.

[97] Amano H., Sakamoto H., Shiga N., Suzuki K. (2016). Method for rapid screening analysis of Sr-90 in edible plant samples collected near Fukushima, Japan. Appl. Radiat. Isot..

[98] Kankia B.I., Marky L.A. (2001). Folding of the thrombin aptamer into a G-quadruplex with Sr^2+^: Stability, heat, and hydration. J. Am. Chem. Soc..

[99] Leung K.-H., Ma V.P.-Y., He H.-Z., Chan D.S.-H., Yang H., Leung C.-H., Ma D.-L. (2012). A highly selective G-quadruplex-based luminescent switch-on probe for the detection of nanomolar strontium(II) ions in sea water. RSC Adv..

[100] Feng L., Wang H., Liu T., Feng T., Cao M., Zhang J., Liu T., Guo Z., Galiotis C., Yuan Y. (2023). Ultrasensitive and highly selective detection of strontium ions. Nat. Sustain..

[101] He W., Li G., Ma X., Wang H., Huang J., Xu M., Huang C. (2006). WEEE recovery strategies and the WEEE treatment status in China. J. Hazard. Mater..

[102] Recknagel S., Radant H., Kohlmeyer R. (2014). Survey of mercury, cadmium and lead content of household batteries. Waste Manag..

[103] Jaishankar M., Tseten T., Anbalagan N., Mathew B.B., Beeregowda K.N. (2014). Toxicity, mechanism and health effects of some heavy metals. Interdiscip. Toxicol..

[104] Zhu Q., Liu L., Xing Y., Zhou X. (2018). Duplex functional G-quadruplex/NMM fluorescent probe for label-free detection of lead (II) and mercury (II) ions. J. Hazard. Mater..

[105] Chen F., Lu Q., Huang L., Liu B., Liu M., Zhang Y., Liu J. (2021). DNA triplex and quadruplex assembled nanosensors for correlating K+ and pH in lysosomes. Angew. Chem. Int. Ed..

[106] Sun J., Lu Y., He L., Pang J., Yang F., Liu Y. (2020). Colorimetric sensor array based on gold nanoparticles: Design principles and recent advances. Trends Anal. Chem..

[107] Yang X., Yuan L., Xu Y., He B. (2023). Target-catalyzed self-assembled spherical G-quadruplex/hemin DNAzymes for highly sensitive colorimetric detection of microRNA in serum. Anal. Chim. Acta.

[108] Zahra Q.U.A., Luo Z., Ali R., Khan M.I., Li F., Qiu B. (2021). Advances in gold nanoparticles-based colorimetric aptasensors for the detection of antibiotics: An overview of the past decade. Nanomaterials.

[109] Chen S., Yu Y.-L., Wang J.-H. (2018). Inner filter effect-based fluorescent sensing systems: A review. Anal. Chim. Acta.

[110] Ge J., Hu Y., Deng R., Li Z., Zhang K., Shi M., Yang D., Cai R., Tan W. (2020). Highly sensitive microRNA detection by coupling nicking-enhanced rolling circle amplification with MoS2 quantum dots. Anal. Chem..

[111] Campuzano S., Yáñez-Sedeño P., Pingarrón J.M. (2019). Carbon Dots and Graphene Quantum Dots in Electrochemical Biosensing. Nanomaterials.

[112] Lu J., Wang J., Hu X., Gyimah E., Yakubu S., Wang K., Wu X., Zhang Z. (2019). Electrochemical Biosensor Based on Tetrahedral DNA Nanostructures and G-Quadruplexb. Anal. Chem..

[113] Wishart D.S. (2019). Metabolomics for Investigating Physiological and Pathophysiological Processes. Physiol. Rev..

[114] Farrugia G., Szurszewski J.H. (2014). Carbon Monoxide, Hydrogen Sulfide, and Nitric Oxide as Signaling Molecules in the Gastrointestinal Tract. Gastroenterology.

[115] Feng G., Luo X., Lu X., Xie S., Deng L., Kang W., He F., Zhang J., Lei C., Lin B. (2019). Engineering of Nucleic Acids and Synthetic Cofactors as Holo Sensors for Probing Signaling Molecules in the Cellular Membrane Microenvironment. Angew. Chem. Int. Ed..

[116] Bours M.J.L., Swennen E.L.R., Di Virgilio F., Cronstein B.N., Dagnelie P.C. (2006). Adenosine 5′-triphosphate and adenosine as endogenous signaling molecules in immunity and inflammation. Pharmacol. Ther..

[117] Zheng J., Wang Q., Shi L., Peng P., Shi L., Li T. (2021). Logic-Gated Proximity Aptasensing for Cell-Surface Real-Time Monitoring of Apoptosis. Angew. Chem. Int. Ed..

[118] Zhang S., Chen X., Pan S., Tang J. (2021). Endocrine and metabolism. Clinical Molecular Diagnostics.

[119] Del Prato S., Marchetti P., Bonadonna R.C. (2002). Phasic Insulin Release and Metabolic Regulation in Type 2 Diabetes. Diabetes.

[120] Wu Y., Midinov B., White R.J. (2019). Electrochemical Aptamer-Based Sensor for Real-Time Monitoring of Insulin. ACS Sens..

[121] Raveendran M., Lee A.J., Sharma R., Wälti C., Actis P. (2020). Rational design of DNA nanostructures for single molecule biosensing. Nat. Commun..

[122] Seferos D.S., Giljohann D.A., Hill H.D., Prigodich A.E., Mirkin C.A. (2007). Nano-flares: Probes for transfection and mRNA detection in living cells. J. Am. Chem. Soc..

[123] Prigodich A.E., Alhasan A.H., Mirkin C.A. (2011). Selective enhancement of nucleases by polyvalent DNA-functionalized gold nanoparticles. J. Am. Chem. Soc..

[124] Cutler J.I., Auyeung E., Mirkin C.A. (2012). Spherical nucleic acids. J. Am. Chem. Soc..

[125] Kelich P., Jeong S., Navarro N., Adams J., Sun X., Zhao H., Landry M.P., Vukovic L. (2021). Discovery of DNA–Carbon Nanotube Sensors for Serotonin with Machine Learning and Near-infrared Fluorescence Spectroscopy. ACS Nano.

[126] Wang L., Wang X., Wu Y., Guo M., Gu C., Dai C., Kong D., Wang Y., Zhang C., Qu D. (2022). Rapid and ultrasensitive electromechanical detection of ions, biomolecules and SARS-CoV-2 RNA in unamplified samples. Nat. Biomed. Eng..

[127] Gao R., Hao C., Xu L., Xu C., Kuang H. (2018). Spiny Nanorod and Upconversion Nanoparticle Satellite Assemblies for Ultrasensitive Detection of Messenger RNA in Living Cells. Anal. Chem..

[128] Zhang D., Peng R., Liu W., Donovan M.J., Wang L., Ismail I., Li J., Li J., Qu F., Tan W. (2021). Engineering DNA on the surface of upconversion nanoparticles for bioanalysis and therapeutics. ACS Nano.

[129] Chen T., Wu C.S., Jimenez E., Zhu Z., Dajac J.G., You M., Han D., Zhang X., Tan W. (2013). DNA Micelle Flares for Intracellular mRNA Imaging and Gene Therapy. Angew. Chem. Int. Ed..

[130] Cao L., Li C.M., Zhen S.J., Huang C.Z. (2023). A General Signal Amplifier of Self-Assembled DNA Micelles for Sensitive Quantification of Biomarkers. Anal. Chem..

[131] Zhang C., Zhao Y., Xu X., Xu R., Li H., Teng X., Du Y., Miao Y., Lin H.C., Han D. (2020). Cancer diagnosis with DNA molecular computation. Nat. Nanotechnol..

[132] Vicente A.M., Ballensiefen W., Jönsson J.-I. (2020). How personalised medicine will transform healthcare by 2030: The ICPerMed vision. J. Transl. Med..

[133] Wang A.G., Dong T., Mansour H., Matamoros G., Sanchez A.L., Li F. (2018). Based DNA reader for visualized quantification of soil-transmitted helminth infections. ACS Sens..

[134] Mahmud A., Chang D., Das J., Gomis S., Foroutan F., Chen J., Pandey L., Flynn C., Yousefi H., Geraili A. (2023). Monitoring Cardiac Biomarkers with Aptamer-Based Molecular Pendulum Sensors. Angew. Chem..

[135] Williamson P., Piskunen P., Ijäs H., Butterworth A., Linko V., Corrigan D.K. (2023). Signal amplification in electrochemical DNA biosensors using target-capturing DNA origami tiles. ACS Sens..

[136] Zhang Q., Ma S., Liu Z., Zhu B., Zhou Z., Li G., Meana J.J., González-Maeso J., Lu C. (2023). Droplet-based bisulfite sequencing for high-throughput profiling of single-cell DNA methylomes. Nat. Commun..

